# The human amygdala in threat learning and extinction

**DOI:** 10.1126/sciadv.aea8233

**Published:** 2026-03-25

**Authors:** Sjoerd Meijer, Eleonora Carpino, Benjamin R. Kop, Jesse Lam, Lycia D. de Voogd, Karin Roelofs, Lennart Verhagen

**Affiliations:** ^1^ Donders Institute for Brain, Cognition and Behavior, Radboud University, Nijmegen, Netherlands.; ^2^ Department of Experimental Psychology, University of Oxford, Oxford, UK.; ^3^ Brain Research and Imaging Centre, Faculty of Health, University of Plymouth, Plymouth, UK.; ^4^ School of Psychology, Faculty of Health, University of Plymouth, Plymouth, UK.; ^5^ Institute of Psychology, Leiden University, Leiden, Netherlands.; ^6^ Leiden Institute for Brain and Cognition, Leiden University, Leiden, Netherlands.; ^7^ Behavioral Science Institute (BSI), Radboud University, Nijmegen, Netherlands.

## Abstract

Here, we resolve the long-standing but unconfirmed hypothesis that the human amygdala is essential for rapidly acquiring cued-conditioned threat responses. We provide causal evidence for the amygdala’s contribution to forming threat memories that are resistant to extinction. Using transcranial ultrasound stimulation (TUS), a noninvasive technique that modulates deep brain structures with high spatial and temporal precision, we targeted the bilateral amygdala during Pavlovian threat conditioning in healthy adults. Linear mixed-effects models and computational modeling of trial-level skin conductance responses revealed that amygdala-TUS (experiment I, *n* = 25), but not hippocampus-TUS (experiment II, *n* = 25), selectively slowed initial threat acquisition, augmented subsequent extinction, and modulated declarative memory of retrospective threat probability. These findings demonstrate that the human amygdala drives an emotional learning state—learning fast, forgetting slow. Our study shows the potential of TUS for targeted neuromodulation of human deep brain structures implicated in conditions such as posttraumatic stress disorder, where pathological threat memories persist despite therapy.

## Teaser

Ultrasonic neuromodulation of the human amygdala provides causal evidence for its role in forming persistent threat memories.

## INTRODUCTION

When memories are formed under threat, they often persist and remain resistant to extinction (*1*–*3*). This poses a central challenge in anxiety- and trauma-related disorders (*4*–*7*). Although animal research suggests a critical role of the amygdala in threat learning and extinction (*8*), causal evidence in healthy humans is lacking, hampering progress in our understanding of threat memories as well as the development of improved treatment. Here, we leverage recent innovations in noninvasive deep brain neuromodulation to causally test whether the human amygdala contributes to learning and subsequent extinction of those threats.

Pavlovian threat conditioning serves as the main experimental model for studying threat learning in both animal and human research (*9*–*12*). In threat conditioning, a neutral stimulus acquires threat value through repeated pairing with an aversive outcome, such as a mild shock. Animal research using this model has consistently identified the amygdala, particularly the basolateral complex, as a critical deep brain structure supporting the rapid acquisition and long-term expression of cued-conditioned threat responses (*13*–*17*). However, the causal contribution of the amygdala to Pavlovian threat learning in humans remains unconfirmed and is an active topic of debate (*18*–*20*). Human neuroimaging studies have yielded mixed results (*18*, *19*, *21*–*27*), and evidence from patients with amygdala lesions is sparse and marred by long-term plastic reorganization (*28*–*30*).

Transcranial ultrasound stimulation (TUS) provides the opportunity for noninvasive, spatially and temporally precise modulation of deep brain structures in humans, including the amygdala (*31*–*33*), through focused delivery of low-intensity ultrasound waves (*34*–*37*). In this study, we selectively targeted bilateral amygdala function during Pavlovian threat conditioning in healthy adults, applying TUS during conditioned stimulus (CS) presentation. We quantified threat and extinction learning through trial-level skin conductance responses (SCRs) and fitted model-free reinforcement learning models (*38*, *39*). We verified successful Pavlovian threat conditioning and established a baseline for assessing neuromodulatory effects through within-session sham conditions. To further confirm the neural specificity of these effects, we conducted a second experiment targeting the posterior hippocampus—chosen on the basis of evidence that its role is selective for trace and contextual, but not cued delayed, threat learning (*40*–*45*). We incorporated the dual sham and hippocampal control stimulation to distinguish between target-specific and general neuromodulation effects, thereby supporting precise causal inferences (*46*).

Across two tightly controlled experiments, we demonstrate that human amygdala function is critical for rapidly acquiring cued-conditioned threat responses. Amygdala-TUS selectively interferes with early threat learning and augments subsequent extinction, establishing a causal link between amygdala function during acquisition and the persistence of associative threat memories. These findings provide direct causal evidence that the human amygdala imposes an emotional learning state—learning fast, forgetting slow.

## RESULTS

### Temporal dynamics of threat learning and extinction in the baseline sham conditions

**Fig. 1.**
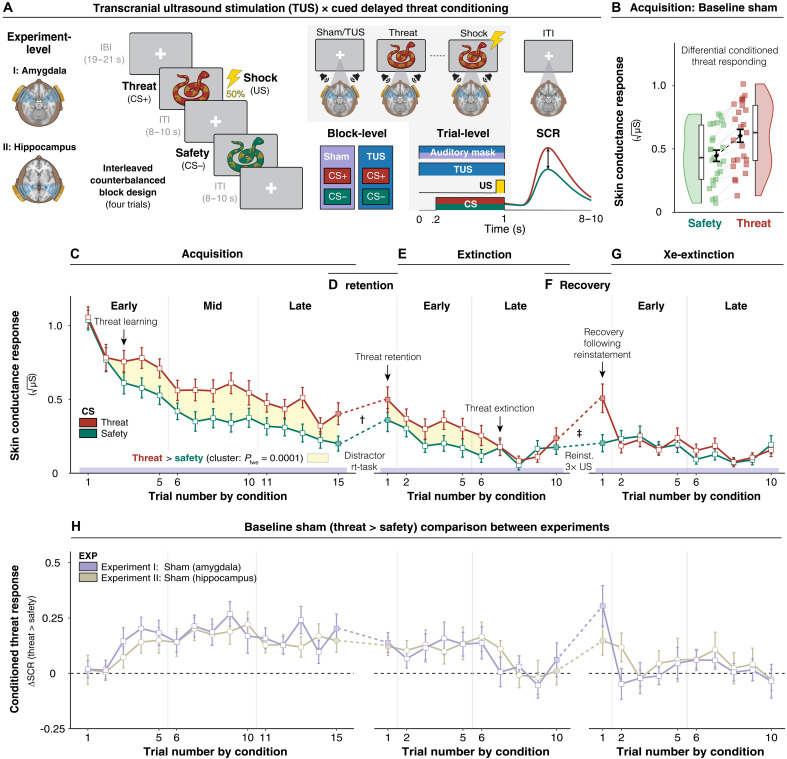
Threat and extinction learning under the baseline sham conditions. (**A**) Pavlovian conditioning task schematic. Left: Threat cues (CS+) were paired with an aversive electric shock (US) on 50% of acquisition trials; safety cues (CS−) were never paired. Middle: Two cue sets were presented in counterbalanced blocks of four trials each, paired with either active TUS (amygdala/hippocampus) or sham stimulation. Each trial consisted of active TUS or sham stimulation (auditory matching stimulus only), initiated 200 ms before CS (snake image) onset, and continued throughout the 800-ms CS presentation and potential US delivery (final 100 ms of reinforced trials). This timing ensured that TUS covered the entire cue-outcome association interval. Intertrial intervals (ITIs) were jittered between 8 and 10 s to allow recovery of electrodermal activity to baseline. Right: Example phasic SCR. (**B**) Differential acquisition responses. Mean SCRs to safety (green) and threat (red) across participants (squares) and group mean (black diamonds, ±SEM; experiment I, *n* = 25). Boxplots and rainclouds display distribution and density. (**C** to **G**) Learning dynamics across Pavlovian conditioning procedures. Trial-by-trial SCRs to threat (red) and safety (green). Safety responses are averaged over adjacent trial pairs to match the number of unreinforced threat trials. Arrows indicate key conditioning effects. Yellow-shaded segments mark clusters where threat > safety [cluster-based permutation, 10,000 permutations, paired one-tailed *t* tests, |*t*| > *q*_0.90_]. Dashed lines denote task breaks: distractor between acquisition and extinction; reinstatement shocks between extinction and re-extinction. (D) Threat memory retention effect (†main CS effect). (F) Recovery following reinstatement effect (‡CS × reinstatement interaction). (**H**) Sham comparison across experiments. Differential SCRs (threat > safety) under sham for amygdala (purple) and hippocampus (beige) groups (±SEM; *n* = 25 each). Sham data from experiment I mirror (C) to (G) for direct comparison. Illustration credit: S. Farbout.

**Fig. 2.**
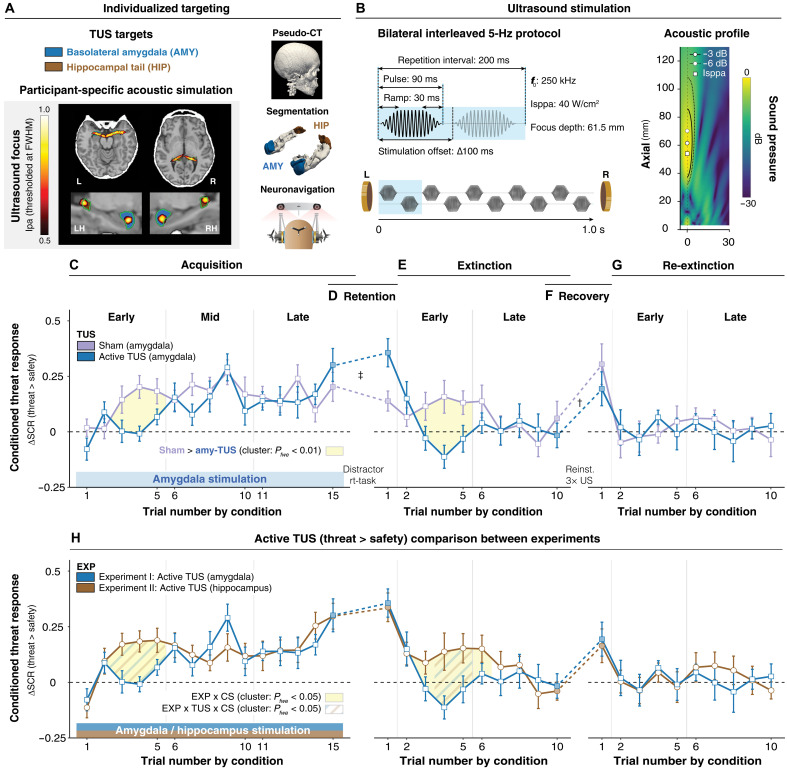
Amygdala-TUS modulation of threat learning and extinction. (**A**) Individualized targeting. Left: Acoustic simulations illustrating the individualized ultrasound focus for basolateral amygdala (blue) and hippocampal tail (brown), shown as normalized intensity profiles at −3 dB [full width at half maximum (FWHM]. Right (top-bottom): Pseudo-CT derived from ultrashort echo-time (UTE) magnetic resonance imaging scans using the open-source PRESTUS toolbox (https://zenodo.org/records/15095861); individualized anatomical segmentations of basolateral amygdala [blue; (*98*)] and hippocampal tail [brown; (*99*)] generated from high-resolution structural T1- and T2-weighted scans using FreeSurfer (*100*); neuronavigated targeting and real-time tracking of bilateral ultrasound transducers. (**B**) Stimulation protocol and free-field ultrasound measurements. Left: Schematic of the interleaved bilateral stimulation protocol designed to maximize acoustic energy delivery while avoiding constructive interference between overlapping foci. Pulses were ramped using a Tukey window on pressure (30 ms on/off) to minimize auditory artifacts. Right: Free-field hydrophone measurements characterizing the acoustic intensity distribution of a single 250-kHz transducer with focal depth setting at 61.5 mm. Two-dimensional ultrasound pressure field with −3-dB (solid) and −6-dB (dashed) contours along the longitudinal axis. The spatial peak pressure (white square; max. pressure = 1.24 MPa; *I*_sppa_ = 38.43 W/cm^2^) and volume centers at −3 dB (white circle; 61.5 mm) and −6 dB (white circle with dashed outline; 70.2 mm) are displayed. (**C** to **G**) Within-amygdala experiment effects. Trial-by-trial mean threat > safety differences under sham and active TUS (±SEM). Yellow-shaded segments mark significant CS × TUS clusters (cluster-based permutation test, 10,000 permutations, paired one-tailed *t* tests, |*t*| > *q*_0.90_). †TUS main effect during threat retention; ‡reinstatement main effect (CS × reinstatement) during recovery following reinstatement (filled symbols). (**H**) Between-experiment effects. Blue, amygdala-TUS; brown, hippocampus-TUS. Trial-by-trial SCR differences (±SEM). Yellow shading marks clusters with significant Experiment × CS interactions; blue-brown dashed segments denote clusters with significant Experiment × CS × TUS three-way interactions.

We investigated the causal role of the human amygdala in threat learning and extinction by combining a classical Pavlovian threat conditioning paradigm with bilateral TUS targeting the amygdala versus sham (experiment I, *n* = 25) or the hippocampus versus sham (experiment II, *n* = 25). Participants were instructed to learn which visual snake cues were associated with a mild aversive shock (Fig. 1A). The threat cue (CS+) was paired with the unconditioned shock stimulus (US) in 50% of the trials. In contrast, the safety cue (CS−) was never paired with the US. Phasic SCRs were used as a well-validated and robust trial-level index of conditioned responding (*47*). Stimulation effects were evaluated by pairing one set of CSs (CS+ and CS−) with active TUS, while a second set was paired with sham stimulation. This design establishes a within-participant sham baseline for assessing the neuromodulatory impact of amygdala-TUS (active stimulation) and hippocampus-TUS (control stimulation) (Fig. 2, A and B).

Following sham stimulation in experiment I, participants rapidly acquired cued-conditioned threat responses (threat > safety; Fig. 1, B and C), reliably expressed threat memory retention (Fig. 1D), with threat responses persisting for several unreinforced threats before extinction (Fig. 1E), and demonstrated recovery of threat responses following three unexpected US presentations (Fig. 1F) and rapid re-extinction (Fig. 1G). This pattern, also observed in the sham condition of experiment II (Fig. 1H), confirms effective Pavlovian threat conditioning and provides a robust baseline against which to test the effects of TUS in both experiments (see also the Supplementary Materials, section S1 and figs. S1 and S2).

### Amygdala-TUS interferes with early threat learning

Amygdala-TUS (Fig. 2, A and B) selectively disrupted early threat learning (early: *t*_24_ = −2.98, *P* = 0.0065, Cohen’s *d* = −0.60; mid: *P* = 0.4170; late: *P* = 0.7355), as evidenced by slower acquisition of conditioned threat responses compared with sham (CS × TUS × TRIAL interaction: *F*_1,52_ = 4.73, *P* = 0.0342, η_p_^2^ = 0.08; sham > amy-TUS: trials 3 to 5, *p*_fwe_ = 0.009; Fig. 2C). The effect of amygdala-TUS versus sham on conditioned responding was primarily driven by slower learning about the threat cue (*t_24_* = −2.81, *P* = 0.0098, Cohen’s *d* = −0.57) relative to safety cue (*P* = 0.2618). These findings align with human neuroimaging studies that show robust amygdala involvement (CS+ > CS−) during early, but not late, stages of conditioning (*19*). Direct comparison of amygdala-TUS and hippocampus-TUS experiments confirmed the target and temporal selectivity of amygdala-TUS effects, which were specific to early acquisition (CS × TUS × EXP interaction: early: *F*_1,48_ = 8.35, *P* = 0.0058, η_p_^2^ = 0.15; mid: *P* = 0.8196; late: *P* = 0.3672; acquisition cluster: trials 3 to 5, *p*_fwe_ = 0.0139; Fig. 2H).

In line with preregistered hypotheses, TUS modulated threat acquisition; however, contrary to the expected gradual evolution of effects with a peak during late learning, the strongest modulation occurred during early acquisition—precisely when amygdala engagement is typically most robust in human neuroimaging (*19*, *20*). This pattern suggests that TUS selectively interfered with amygdala-dependent components of early learning, without globally impairing the ability to acquire threat associations (CS × TUS × TRIAL × EXP interaction: *P* > 0.05).

### Associative threat memories acquired during amygdala-TUS extinguish faster

TUS was discontinued after acquisition to assess whether threat memories formed under amygdala-TUS are more susceptible to extinction. Our results show that amygdala-TUS during acquisition accelerates subsequent early extinction (early: *t*_24_ = −2.81, *P* = 0.0097, Cohen’s *d* = −0.56; late: *t*_24_ = −0.59, *P* = 0.6530), evidenced by a faster decay in conditioned threat responding, with participants reaching safety cue levels several trials earlier than in the sham condition (sham > amy-TUS: trials 3 to 5, *p*_fwe_ = 0.0049; Fig. 2E). In accordance with the cue-specific effects observed on threat acquisition, amygdala-TUS effects on extinction were strongest for the threat cue (*t_24_* = −2.34, *P* = 0.0280, Cohen’s *d* = −0.47) compared with the safety cue (*t_24_* = 0.95, *P* = 0.3497). These findings suggest that the observed amygdala-TUS effect on threat learning results in a less stable and more readily extinguished threat memory trace. Consistent with the acquisition phase, direct comparison of the amygdala-TUS and hippocampus-TUS experiments confirmed the target and temporal selectivity of amygdala-TUS effects on conditioned responding during extinction, with effects restricted to early extinction (CS × TUS × EXP interaction: early: *F*_1,96_ = 4.74, *P* = 0.0320, η_p_^2^ = 0.05; late: *P* = 0.8781; extinction cluster: trials 4 and 5, *p*_fwe_ = 0.0139; Fig. 2H).

### Amygdala-TUS effects are specific to learning new associations

Next, we examined whether amygdala-TUS specifically affects new associative learning or also affects the expression of previously formed threat and extinction memories. We observed no effects of amygdala-TUS on re-extinction (early: *t*_24_ = 0.46, *P* = 0.6472; late: *t*_24_ = 0.40, *P* = 0.6965; Fig. 2G), threat retention (*F*_1,120_ = 0.15, *P* = 0.7029; Fig. 2D), or recovery following reinstatement (*F*_1,120_ = 0.06, *P* = 0.8102; Fig. 2F). These findings highlight the specificity of amygdala-TUS to associative memory formation and updating, rather than memory retention or expression.

Contrary to our preregistered prediction, stimulation during threat acquisition did not affect later threat retention or recovery following reinstatement compared to sham. As participants reached comparable asymptotic levels of conditioned responding by the end of acquisition and extinction, subsequent threat retention and recovery may reflect amygdala-independent memory expression and learning, consistent with reduced amygdala engagement during these later phases in human neuroimaging studies (*19*, *20*). We conducted additional control analyses, which confirm that amygdala-TUS does not affect reinforced threat responses or habituation (all *P* values > 0.05; Supplementary Materials, section S2).

### Amygdala-TUS modulates declarative memory of threat probability

**Fig. 3.**
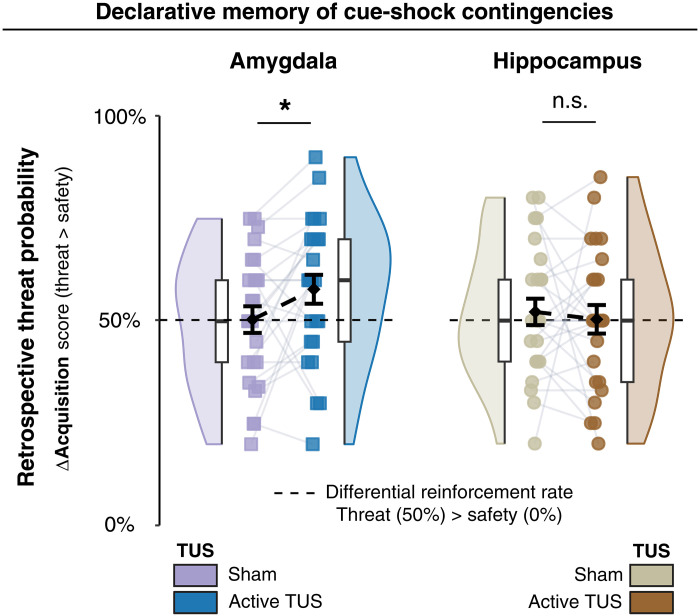
Amygdala supports accurate threat prediction based on postexperimental memory recollection of threat experience. Differential retrospective threat probability ratings (threat > safety) collected after task completion. Colors indicate TUS condition (active versus sham) within each experiment. The dashed line at 50% denotes the experimentally defined differential reinforcement rate [50% shock (US) probability for the threat cue (CS+), 0% for the safety cue (CS−)] and serves as a reference for accurate recollection of threat contingencies. Individual participant ratings are shown as squares (experiment I: purple, sham; blue, active TUS; experiment II: beige, sham; brown, active TUS). Black diamonds indicate group means (±SEM), and overlaid boxplots and rainclouds depict distribution and density. The asterisk (*) denotes a significant main effect of TUS on differential ratings in experiment I (*P* < 0.05). In experiment II, no significant TUS effects were observed. n.s., not significant.

Retrospective subjective ratings, collected following task completion, revealed that participants who received amygdala-TUS overestimated retrospective threat probability associated with the threat versus safety cue (*t*_24_ = 2.14, *P* = 0.0427, Cohen’s *d* = 0.43; Fig. 3), without affecting valence or arousal scores (all *P* values > 0.05; Supplementary Materials, section S3 and fig. S1F). This suggests that normal amygdala function supports precise threat prediction and that disrupting this process leads to less calibrated associative memory representations.

### Hippocampus-TUS confirms target specificity of amygdala-TUS effects

To verify anatomical specificity, we performed a second experiment targeting the hippocampus as an active control site. Conditioned threat responses under hippocampus-TUS did not differ significantly from sham (all *P* values > 0.05; Supplementary Materials, section S4), confirming robust task performance.

Across both experiments, the SCRs in the last trial of acquisition and the first trial postacquisition (threat retention) were higher in both active TUS conditions (amygdala-TUS and hippocampus-TUS) compared with sham (Fig. 2H; Supplementary Materials, section S5). This observation likely reflects a nonspecific effect of TUS, perhaps multimodal cueing, and thus underscores the importance of active control conditions for isolating target-specific neuromodulatory effects from nonspecific global influences of stimulation. Subjective peripheral sensations and placebo-related beliefs associated with stimulation did not differ between experiments (all *P* values > 0.05; Supplementary Materials, section S5), further supporting their comparability for assessing target specificity. Collectively, these findings show that the effects of amygdala-TUS on threat learning and extinction are both temporally and target specific. To determine whether the TUS effects on conditioned responding observed in the linear mixed-effects models and permutation analyses reflected changes in underlying learning mechanisms, we next applied hierarchical reinforcement learning models to the SCR data.

### Amygdala-dependent emotional learning state—Learning fast, forgetting slow

**Fig. 4.**
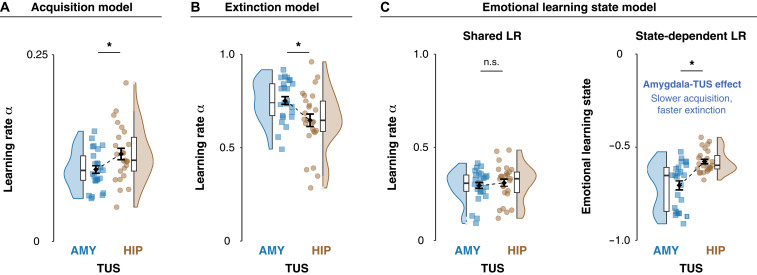
Amygdala-TUS versus hippocampus-TUS modulates emotional learning state during threat acquisition and extinction. (**A**) Acquisition learning rates. Individual learning rates (α) were estimated using a hierarchical Rescorla-Wagner model fit to trial-level SCRs during acquisition. Blue squares (*n* = 25) show participants receiving amygdala-TUS; brown circles (*n* = 25) show hippocampus-TUS. Black diamonds indicate group means (±SEM). Boxplots and rainclouds display the underlying distribution and density. (**B**) Extinction learning rates. Individual learning rates from the extinction phase. Amygdala-TUS participants (blue) exhibit higher extinction rates than hippocampus-TUS participants (brown), indicating accelerated updating of threat memories during extinction. (**C**) Emotional learning state model. Left: Shared learning rate across acquisition and extinction shows no overall difference between active amygdala-TUS and hippocampus-TUS, confirming that base learning capacity is comparable. Right: Context-dependent modulation of emotional learning state under amygdala-TUS reveals a reversal of the typical bias—shifting from rapid acquisition and slow forgetting toward slower learning and faster extinction. Asterisks (*) denote a significant main effect of TUS (*P* < 0.05).

Computational modeling of threat learning behavior revealed that amygdala-TUS, compared with hippocampus-TUS, selectively reduced learning rates during threat acquisition (*t*_48_ = −2.29, *P* = 0.0263; Fig. 4A), impaired threat value updating following shock delivery (Supplementary Materials, section S6), and increased learning rates during extinction (*t*_48_ = 2.68, *P* = 0.0101; Fig. 4B). To comprehensively consider acquisition and extinction in a singular integrative framework, we implemented an emotional learning state model. In this model, a singular learning rate, shared across acquisition and extinction, is differentially modulated by a context-specific parameter, depending on whether participants are in a threat (acquisition) or safety (extinction) state. This model parsimoniously captures both the immediate and downstream effects of amygdala-TUS across learning phases. Critically, it revealed a robust reduction in emotional learning bias following amygdala-TUS versus hippocampus-TUS (*t*_48_ = −4.73, *P* < 0.0001; Fig. 4C), while TUS left the shared learning rate unaffected (*t*_48_ = −0.5787, *P* = 0.5655). These results support the interpretation that amygdala activity enacts a persistent emotional learning state—learning fast, forgetting slow—which underlies the rapid formation of threat memories and their susceptibility to extinction.

## DISCUSSION

This study provides causal evidence for amygdala involvement in threat and extinction learning in healthy humans. We used TUS to noninvasively modulate the bilateral amygdala with high temporal and spatial precision during Pavlovian threat conditioning. Amygdala-targeted interference during acquisition slowed early threat learning and accelerated subsequent extinction compared with sham and hippocampal control stimulation. These observations confirm and extend hypotheses derived from animal lesion studies (*48*–*52*) and contribute to resolving ongoing debates in human neuroimaging that challenge amygdala involvement in threat learning (*18*). We propose a conceptual framework for amygdala function in cued associative threat learning, whereby the human amygdala drives an emotional learning state—learning fast, forgetting slow. Our study highlights the potential of TUS for noninvasive amygdala modulation in humans and may pave the way for future amygdala-targeted interventions in anxiety- and trauma-related disorders.

### Amygdala-dependent threat associations resist extinction

We observed that without active amygdala stimulation, whether sham or hippocampus stimulation, participants rapidly acquired conditioned threat responses, differentiating between threat and safety cues within a few trials (Figs. 1, C and H, and 2D). In contrast, with amygdala-TUS, the emergence of conditioned threat responses slowed precisely during this critical window (Fig. 2, A and F), confirming that amygdala engagement is essential for rapid initial threat learning (*53*).

These temporally selective effects clarify mixed findings in human neuroimaging regarding amygdala activation during Pavlovian threat conditioning. Some research shows robust amygdala activation during acquisition (*19*, *21*–*24*), while others note minimal or no activation (*18*, *25*–*27*). Such inconsistencies are thought to result, in part, from variability in the timing and duration of amygdala responses (*19*, *20*). Our findings bolster the argument for a time-critical function of the amygdala and underscore the potential of noninvasive neuromodulation for causal testing of contrasting hypotheses derived from human neuroimaging.

During early acquisition, when individuals are still learning which cues are paired with an aversive shock, the unexpected delivery of shock results in large prediction errors (PEs), driving rapid Pavlovian learning (*38*, *39*). Here, using computational modeling of trial-level SCRs, we demonstrate that amygdala-TUS reduced individuals’ acquisition learning rates, indicating slower updating of threat value following aversive outcomes, an effect not observed under hippocampus-TUS (Fig. 4A). Further, we demonstrate that the amygdala selectively modulates conditioned responding on trials following shock delivery (fig. S3). This finding supports the perspective that the amygdala updates threat associations when cues and unexpected reinforcers occur together in time (*54*). It also indicates that this process can be disrupted by applying TUS during this critical window.

One key question is whether the amygdala is responsible for storing threat memories or whether it primarily facilitates their consolidation and retrieval through connected circuits (*8*). Our experimental design might provide insight into this debate. Permanent amygdala lesions in animals disrupt both threat learning and extinction, leaving unresolved whether extinction impairments reflect deficient acquisition or the absence of amygdala function during extinction itself. By contrast, by applying transient and temporally precise modulation during acquisition, we demonstrate that amygdala interference at the time of learning leads to downstream effects on extinction. Without active amygdala stimulation, threat responses persisted over several unreinforced threats (Fig. 1, E and H), highlighting the extinction resistance imposed by the partial reinforcement schedule, i.e., partial reinforcement extinction effect (*55*). By contrast, amygdala-TUS during acquisition resulted in accelerated extinction, reaching safety cue levels significantly faster (Fig. 2, C and H). Computational modeling confirmed increased learning rates during extinction when acquisition had been disrupted by amygdala-TUS (Fig. 4B). These findings show that amygdala-dependent threat learning affects the susceptibility of threat memories to extinction.

### Learning systems supporting fast versus slow threat learning

Notably, individuals undergoing amygdala-TUS eventually acquired threat and safety contingencies, albeit more slowly and with less persistent threat memories (Fig. 2). One might argue that this observation contradicts animal studies involving amygdala lesions, which indicate complete disruption (*49*, *50*). However, several studies highlight that conditioned threat responses can still develop after amygdala lesioning, albeit at a slower pace and requiring extended training (*48*, *51*, *52*). Critically, those amygdala-independent threat memories extinguish faster upon repeated safety exposures (*48*, *51*, *52*). Collectively, these findings from animal models emphasize two distinctive survival-relevant features of amygdala-dependent threat learning—fast acquisition and slow extinction—for which we now provide causal evidence in humans.

We speculate that the preserved capacity for threat learning under amygdala interference may rely on alternate value-based learning systems within the broader threat learning and extinction network (*27*, *56*), such as the ventromedial-orbitofrontal cortex and ventral striatum (*24*, *57*–*61*). Future studies combining TUS with functional neuroimaging or electrophysiology will be critical to establish how modulation of amygdala activity alters large-scale neural dynamics supporting threat learning and extinction.

### Threats engage emotional learning state

How humans learn about threats may affect subsequent extinction. Our paradigm, in which amygdala stimulation was applied exclusively during acquisition, allows us to distinguish between three alternative accounts of amygdala function in Pavlovian cued conditioning: separate, shared, or state-dependent learning. A separate learning account would predict independent modulation of acquisition and extinction learning, which does not align with our observation that amygdala-TUS during acquisition also affects subsequent extinction learning (Figs. 2, C and H, and 4B). Nor do our observations align with a shared learning model, wherein a singular learning state is shared across acquisition and extinction. Under such a model, one might expect the amygdala to encode high cue saliency irrespective of valence, enhancing learning similarly for both acquisition and extinction. In contrast, we observed that amygdala-TUS during acquisition had downstream effects on both acquisition (Fig. 4A) and extinction (Fig. 4B). Thus, a parsimonious computational account of the observed bidirectional impact of amygdala-TUS requires modulation of learning dependent on the current emotional state (acquisition/extinction). This emotional state-dependent learning model assumes that initial affective tagging of threat associations affects subsequent extinction learning. Fitting this model to the observed behavior confirmed a shift from fast learning and slow forgetting under hippocampus-TUS to slower learning and faster forgetting following amygdala-TUS (Fig. 4C). This model echoes earlier suggestions for the role of the amygdala in enacting a motivational state on learning systems (*62*) and expands this to emotional learning state in humans (*63*, *64*).

### Limitations and future outlook

Noninvasive deep brain neuromodulation with TUS offers causal precision in human cognitive neuroscience that was previously accessible only through invasive approaches or animal models. TUS complements the hypothesis-generating and circuit-level perspective of correlational neuroimaging (*65*) and addresses some of the challenges of translating from lesion models—with altered neural plasticity and reorganization—to healthy temporal and functional specificity (*66*, *67*). Here, we have focused on causal amygdala contributions to threat learning and extinction, but this approach could be expanded to other emotional and cognitive domains, such as decision-making under uncertainty, reward processing, or cognitive control, which critically depend on deep brain function.

Our findings provide causal evidence that phasic SCRs index amygdala-dependent associative learning in cued Pavlovian threat conditioning, consistent with proposals that SCRs track current threat value or associability during learning (*68*). While concurrent neuroimaging could provide a more direct estimate of neural target engagement, our conclusions draw on the use of a well-established threat conditioning paradigm and tight experimental controls to infer both functional and target specificity. Future studies integrating TUS with functional neuroimaging or electrophysiology will be essential to link local and circuit-level neuromodulation to behavioral outcomes (*35*).

We implemented a single bilateral, interleaved stimulation protocol designed to transiently interfere with amygdala function. However, as with other noninvasive neuromodulation techniques (*69*, *70*), the direction and magnitude of TUS effects likely depend on specific stimulation parameters and the neural state at the time of application (*71*, *72*). Consequently, the present pattern of behavioral effects may be specific to the parameters, target, and behavioral task used here. These parameters may also influence peripheral sensations and placebo-related beliefs (*73*–*75*), which in the present study were matched by targeting the hippocampus as an active control site. However, we did not assess blinding efficacy or perceived sensations associated with stimulation as a function of individual stimulus cues, which we recommend for future studies to further strengthen within-participant comparisons of active versus sham stimulation. Further, systematically mapping both the parameter space and the neural state space could help guide optimization of pulsing regimes to achieve intended neural and behavioral outcomes (*76*, *77*). To enable accurate mapping of dose-response relationships, it would be valuable to combine these approaches with in vivo exposure measurements to account for individual variability in targeting and transmission efficiency (*36*, *37*, *78*).

Here, amygdala-TUS was applied during threat acquisition, which augmented subsequent extinction. For clinical translation, an important next step will be to test whether amygdala-TUS can also facilitate extinction when applied after threat memories have been established (*79*). Promising therapeutic avenues include precisely timed neuromodulation during amygdala-dependent reconsolidation windows, when previously learned threat memories are expected to be susceptible to updating (*80*, *81*). Alternatively, repetitive TUS protocols could be used to induce transient states of heightened plasticity to facilitate extinction learning (*3*, *35*, *82*). Deep brain stimulation (DBS) of the amygdala has demonstrated preliminary efficacy in severe and treatment-resistant posttraumatic stress disorder (*83*). TUS may build on and extend these advances by enabling noninvasive, spatially precise, and temporally controlled modulation of amygdala activity (*34*), paving the way for scalable and mechanistically informed interventions.

Collectively, our findings provide causal evidence supporting a conceptual framework in which the amygdala drives a sustained emotional learning state—rapidly acquiring threat associations that resist extinction. By elucidating how threats engage this emotional state and how it can be modulated through targeted neural interventions, we clarify long-standing debates on human amygdala function and bridge translational gaps between animal models and human neuroscience. This framework advances our theoretical understanding of amygdala computations in the context of threat and may open previously unexplored avenues for precision neuromodulation treatments in anxiety- and trauma-related disorders characterized by maladaptive threat memories.

## MATERIALS AND METHODS

### Participants

This study was preregistered on the Open Science Framework (OSF; https://doi.org/10.17605/OSF.IO/EWKXM). The target sample size was determined on the basis of an a priori power analysis. Given the absence of prior studies combining amygdala-TUS with Pavlovian threat conditioning, the expected effect size was informed by previous behavioral TUS work (*84*–*86*) and human threat conditioning studies reporting small-to-moderate effects (*87*, *88*). On the basis of these considerations, we powered a mixed-design analysis of variance (ANOVA) targeting the TUS × Experiment interaction on differential conditioned responding (CS+ > CS−) to detect a small effect size (*f* = 0.2) with 80% power at α = 0.05, yielding a total of *n* = 52 participants [*n* = 26 per experiment; G*Power v3.1; (*89*)]. This interaction provides a stringent test of TUS effects, as it captures both within-participant (active versus sham TUS) and between-participant (amygdala versus hippocampus target) variance. Using the differential (CS+ > CS−) score further provides a conservative approximation of the preregistered mixed-effects analyses, which model CS+ and CS− jointly and thus offer greater sensitivity. Together, this approach aimed to ensure an appropriately powered design under conservative assumptions, allowing us to detect within-experiment TUS effects and assess their target specificity across experiments.

Before participation, a structured interview was conducted by a trained experimenter to screen for exclusion criteria. We excluded individuals with a history of brain surgery, serious head trauma, epilepsy, seizures or convulsions, diagnosed neurological or psychiatric disorders, or the presence of metal in the head or upper body. Additional exclusion criteria included consumption of more than four alcoholic units within the 24 hours before the session or any recreational drug use within the previous 48 hours. To screen for specific phobias, participants also completed the self-report Snake Anxiety Questionnaire [SNAQ-12; (*90*)]. Those meeting the criteria for snake phobia were excluded. A total of 50 participants (*n* = 25 per experiment) were included in the final analysis (experiment I: *M*_age_ = 24.6, ± SD_age_ = 2.3, range = 19 to 29; 16 females; experiment II: *M*_age_ = 23.7, ± SD_age_ = 1.7, range = 18 to 26; 18 females).

All participants provided written informed consent in accordance with the Declaration of Helsinki, and the study was approved by the local ethics committee (CMO2021-8238, Commissie Mensgebonden Onderzoek Arnhem-Nijmegen). In each experiment, one participant was excluded on the basis of the following data exclusion criterion: failure to show evidence of differential threat conditioning in the baseline sham condition. Specifically, participants were excluded if their average SCR to the threat cue during acquisition was not greater than their response to the safety cue. This criterion was preregistered.

### Study overview

This study used a mixed design, with data collected across two separate sessions for each participant. Session 1 consisted of structural and functional magnetic resonance imaging (MRI) scanning. Session 2 was conducted on a separate day in a dedicated noninvasive brain stimulation laboratory, where participants received the TUS intervention during Pavlovian threat conditioning along with physiological measurements.

During the second session, participants were first prepared for physiological recording, which included the placement of electrodes for physiological measurements and the delivery of shocks. This was followed by a stepwise titration procedure to determine the individual tolerable shock level. We used neuronavigation based on individual structural MRI scans to target TUS. Participants subsequently performed a Pavlovian threat conditioning task lasting ~55 min, during which TUS was administered simultaneously. Throughout the task, physiological responses were recorded, including SCRs.

Following completion of the Pavlovian conditioning paradigm, participants completed a structured debriefing with a trained experimenter. First, we assessed potential sensory experiences and placebo-related beliefs associated with receiving TUS. Participants were asked whether they thought they had received real or placebo (inactive) ultrasound stimulation. We also asked about possible auditory or somatosensory sensations (“Could you hear or feel the ultrasound stimulation?”). The latter responses were subsequently categorized as auditory, tactile, or thermal sensations (*73*). Second, for each of the four CSs, participants provided subjective ratings of valence (1 = very positive to 7 = very negative), arousal (1 = very calm to 7 = very aroused), and retrospective threat probability (0 to 100%; “Based on your experience during the acquisition phase, how likely was it that you received a shock with this snake?”), with instruction to base their responses on the acquisition phase. These ratings were collected once after task completion and were not assessed on a trial-by-trial basis.

The experiment was conducted under a partially double-blinded design. Participants were blinded to both the stimulation (active TUS versus sham stimulation) and target site conditions (amygdala versus hippocampus), while investigators were blinded to the stimulation condition.

### Magnetic resonance imaging

MRI scanning was performed using a 3-T Magnetom Skyra scanner (Siemens AG, Erlangen, Germany) equipped with a 32-channel head coil. During structural scan acquisition, participants were instructed to lie still with their eyes closed to minimize motion-related artifacts.

A high-resolution T1-weighted (T1w) structural scan was acquired in the sagittal plane [repetition time (TR) = 2700 ms; echo time (TE) = 3.67 ms; flip angle = 9°; voxel size = 0.9 mm by 0.9 mm by 0.9 mm; field of view = 230 mm] for anatomical segmentation and neuronavigation during subsequent TUS sessions. To support whole-head tissue reconstruction and accurately capture skull morphology for acoustic simulations of ultrasonic wave propagation, additional high-resolution structural scans included a T2-weighted (T2w) sequence (sagittal plane; TR = 3200 ms; TE = 408 ms; flip angle = 120°; voxel size = 0.9 mm by 0.9 mm by 0.9 mm; field of view = 230 mm) and a pointwise encoding time reduction with radial acquisition (PETRA) ultrashort TE (UTE) scan (transversal plane; TR = 3.32 ms; TE = 0.07 ms; flip angle = 2°; voxel size = 0.8 mm by 0.8 mm by 0.8 mm; field of view = 294 mm).

### Pavlovian threat conditioning

We investigated the causal role of the human amygdala in threat learning and extinction by combining a classical Pavlovian threat conditioning paradigm with TUS targeting the bilateral amygdala (active target; *n* = 25) or bilateral hippocampus (active control target; *n* = 25) (Fig. 2, A and B). With our current TUS setup, it is not feasible to interleave stimulation of the bilateral amygdala and hippocampus within the same conditioning session. A within-participant design would thus require separate sessions, with concomitant potential learning-history and carry-over effects across sessions. To minimize the risk of these unwanted effects, the two targets were instead tested in separate experiments with independent participant samples.

The experiment consisted of five main phases: threat acquisition (120 trials), threat retention (4 trials), extinction training (40 trials), recovery following reinstatement (4 trials), and re-extinction (40 trials). A short distractor task was presented between the acquisition and the first extinction phase, after which participants were informed that “the snake task will now continue.” During extinction, all CSs (CS+ and CS−) were presented without the US. After the first extinction phase, participants received three unsignaled shocks during the presentation of a white circle to induce reinstatement, followed by a second extinction phase to assess recovery following reinstatement and re-extinction, respectively.

We refer to the first trial after the distractor task as a test of threat retention, used to assess potential sustained effects of TUS on conditioned responding by comparing the last acquisition trial (when TUS is still applied) with the first postacquisition trial (when TUS is no longer applied) for each cue. The first trial after reinstatement served as a test of recovery of conditioned threat responses. We quantified this by comparing differential responding (CS+ > CS−) between the last extinction trial and the first postreinstatement trial. This comparison aims to index the balance between threat and extinction memory traces.

Participants were instructed to learn which visual snake cues [(*91*); Geneva affective picture database (GAPED) images: Sn033, Sn094, Sn123, and Sn131] were associated with a mild aversive shock (“In the following task, your goal is to learn which snakes are paired with a shock”). To ensure that associative learning was experience based, participants received no further contingency instructions.

Each participant viewed four snake cues, organized into two pairs (one CS+ and CS− per pair). One pair was always presented during active TUS and the other during sham stimulation. TUS was applied during both CS+ and CS− presentations to match general, nonspecific stimulation effects (e.g., auditory and somatosensory sensations) across cue types. Our primary inferential test assessed whether active versus sham stimulation altered differential threat learning (CS+ > CS−). To establish cue specificity, we then conducted separate post hoc comparisons (active TUS versus sham) for CS+ and for CS−. This allowed us to determine whether stimulation selectively modulated threat learning as opposed to safety learning. Within each pair, the CS+ was paired with the US on 50% of the trials, and the CS− was never paired with the US. This yielded one threat cue and one safety cue per stimulation condition (CS+_TUS_/CS−_TUS_ and CS+_sham_/CS−_sham_). This 2 by 2 design establishes a within-participant sham baseline for assessing the neuromodulatory impact of amygdala-TUS versus hippocampus-TUS. Phasic SCRs were used to evaluate CS and TUS effects as a well-validated and robust trial-level index of conditioned responding (*47*).

### Trial timing

Each trial began with a 200-ms fixation cross, followed by an 800-ms CS presentation (Fig. 4C). On reinforced CS+ trials, the US was delivered during the final 100 ms of CS presentation. Intertrial intervals were jittered between 8 and 10 s to allow skin conductance to return to baseline. Interblock intervals were jittered between 19 and 21 s to prevent potential stimulation carry-over effects. All stimulus presentation was controlled using PsychoPy [v2021.2.3; (*92*)] and presented on a 24-inch (60.96-cm) monitor with a refresh rate of 120 Hz.

TUS was applied online, concurrent with task performance. Each TUS trial began with TUS onset 200 ms before CS onset and continued for 1000 ms, encompassing the full cue-outcome window (Fig. 1A). This timing ensured that each amygdala received one ultrasound pulse before the presentation of the CS, thereby aiming to control for potential response latency in neuromodulatory effects (*93*).

Accurate stimulation timing was achieved using external triggering via Signal (v7.05; CED, Cambridge, UK) driving a Micro1401 system (CED, Cambridge, UK). TUS parameters were adapted between active and sham TUS blocks using serial commands in PsychoPy [v2021.2.3; (*92*)].

To mask potential auditory artifacts from the stimulation (*75*), an auditory matching sound comprising a 16-kHz sinusoidal waveform tapered by a 10-Hz Tukey envelope (30 ms during pulse on- and offset) with Gaussian white noise (signal-to-noise ratio of 14:1) was played through table speakers on both active TUS and sham stimulation trials. The volume was set uniformly across participants.

### Task structure and stimulation conditions

During the acquisition phase, participants received both active and sham stimulation in a within-participant design. Active stimulation was applied only during acquisition to assess whether modulating amygdala function during threat learning influenced subsequent extinction. During extinction and re-extinction, stimulation was switched to sham to maintain consistent contextual cues across phases.

The threat conditioning task was presented in a pseudorandomized block design with four trials per block to (i) facilitate differential threat learning by presenting the CSs in pairs (active TUS pair: CS+_TUS_, CS−_TUS_; sham stimulation pair: CS+_sham_, CS−_sham_); and (ii) avoid potential carry-over effects from active TUS to sham stimulation trials. There were never more than two consecutive blocks of the same CS pair. In both experiments, the pairing of the four different snake identities (CSs) with TUS conditions was counterbalanced across participants. This was done to prevent potential confounding effects of snake identity on learning. The pseudorandomization included additional constraints to ensure consistent stimulus presentations throughout different experimental phases, supporting comparable learning across participants and experimental conditions over time.

### Shock titration

Before the task, participants underwent a standardized five-step shock titration procedure to determine an individually tolerable but aversive stimulation level (*87*). Shocks were delivered via Ag/AgCl electrodes placed on the distal phalanges of the ring and little fingers of the right hand using a transcutaneous electrical nerve stimulator unit (Prostim 2000, Bio-Protech Inc., Korea). Stimulation consisted of a 100-ms pulse train (150 Hz, 250-μs pulses).

### Distractor task

To introduce a brief delay between the acquisition and extinction phases, participants completed a simple reaction time task lasting ~2 min. On each trial, they viewed a set of four arrows, all pointing in the same direction (left or right), and were instructed to respond as quickly and accurately as possible by pressing the corresponding arrow key. Before the onset of the task, it was explicitly stated that no shocks would be administered. A fixation cross appeared between trials and immediately after each response.

### SCR recording

Electrodermal activity was recorded at 500 Hz from the distal phalanges of the index and middle fingers of the left hand to assess SCRs using a BrainAmp EXG MR 16-channel recording system with BrainVision Recorder (Brain Products GmbH, Gilching, Germany).

### TUS protocol

TUS was delivered using two four-element piezoelectric ultrasound transducers (fundamental frequency, 250 kHz; aperture diameter = 64 mm), driven by a NeuroFUS Pro system (TPO-203-035 for CTX-250-001 and TPO-105-010 for CTX-250-026, Sonic Concepts Inc., Bothell, WA, USA; supplier/support: Brainbox Ltd., Cardiff, UK). Ultrasound was delivered at a spatial-peak pulse-average intensity (*I*_sppa_) in free water of 40 W/cm^2^ at a focal depth of 61.5 mm (Fig. 2B). The pulsing protocol used pulses of 90-ms duration, ramped with Tukey shape on the pressure over 30 ms on/off, repeated at 5 Hz for a total pulse train duration of 1000 ms (Fig. 2B and table S1). This protocol was designed on the basis of prior human studies using 5-Hz protocols in humans in offline stimulation settings (*35*, *94*, *95*) and converging evidence of increased neuromodulatory efficacy when using longer pulses at higher intensities with lower fundamental frequencies (*71*, *72*), including when targeting the nonhuman primate amygdala (*34*). This protocol was applied bilaterally and interleaved across hemispheres to transiently interfere with amygdala function during ongoing threat learning. The estimated exposure adheres to the International Transcranial Ultrasound Stimulation Safety and Standards consortium (ITRUSST) recommendations for biophysical safety [see table S1 for safety metrics; (*96*)]. This protocol was delivered bilaterally, at both amygdalae, whereby the two transducers were sonicating in an interleaved fashion, one delivering ultrasound in the off period of the other. This bilateral 5-Hz TUS protocol was designed to maximize the temporal delivery of energy at both targets while avoiding constructive wave interference patterns. We chose to stimulate the amygdala bilaterally to maximize intervention efficacy, as nonhuman primate work has shown intermediate efficacy of unilateral compared to bilateral amygdala lesions on behavior (*97*). To minimize auditory confounds, we used ramped pulses and played an auditory matching sound through table speakers (*75*).

### Neuronavigation and transducer coupling

The transducers were aligned with the target location and continuously tracked throughout the session using frameless stereotaxic neuronavigation (Localite Biomedical Visualization Systems GmbH, Sankt Augustin, Germany). To maintain stable targeting during the experiment, transducers were secured with FISSO Medical Articulated Arms (Baitella AG, Zurich, Switzerland). Participants rested their heads on a chin rest and back-of-head support, ensuring a consistent head position relative to the transducers.

Individualized targeting of the basolateral amygdala (*98*) or hippocampal tail (*99*) was achieved by extracting the centroid of FreeSurfer segmentations [(*100*); Fig. 2A], based on each participant’s T1- and T2-weighted structural scans. Transducer positions for the target regions were recorded during the experiment and quantified for post hoc simulations of ultrasound wave propagation in k-Plan (*101*).

The transducers were coupled to the participant’s head using in-house developed degassed silicon water balloons attached to the transducer face. Ultrasound gel (Aquaflex Ultrasound Gel, Parker Laboratories) was applied to the participant’s scalp to ensure effective acoustic coupling. All gel application procedures were carefully optimized to minimize air bubbles, adhering to best practices (*102*).

### SCR scoring

We preregistered SCR as the primary outcome parameter to evaluate the neuromodulatory effects of amygdala-TUS versus hippocampus-TUS. We based this on several lines of evidence suggesting that SCRs are amygdala dependent in humans: (i) neuroimaging studies show that amygdala activation during threat learning shows a positive association with measured SCRs (*103*) and with threat learning indices derived from SCRs using computational approaches (*68*); (ii) DBS targeting amygdala in patients with epilepsy results in dose-dependent increases in phasic electrodermal activity (*104*); and (iii) SCRs indexing threat learning are affected by amygdala lesions (*28*).

SCR data were calculated as the trough-to-peak amplitude in electrodermal activity using Autonomate (*105*) and expressed in microsiemens. The parameters for quantifying SCR were determined on the basis of recommendations from the literature (*106*, *107*). Accordingly, we only considered the first response that occurred between 1.0 and 4.0 s after the onset of the CS. The ascent time from the initial deflection to the peak could not exceed 5.0 s. An SCR with an amplitude below 0.01 μS was considered a nonresponse. A square root transformation was used to normalize the SCR data.

### Behavior analyses

Threat learning is inherently dynamic associated with changes in amygdala activation over the course of learning, reflected in the temporal development of differential SCRs (threat > safety) across the Pavlovian conditioning phases (Fig. 1, C to H). To capture these fine-grained learning dynamics, we used three established yet state-of-the-art statistical approaches in the human threat conditioning field: (i) trial-level and time-binned linear mixed-effects models, which capture temporal specificity; (ii) nonparametric permutation-based tests, which assess temporal clusters of condition effects; and (iii) hierarchical reinforcement learning models to infer the latent learning mechanisms underlying these behavioral effects, including acquisition and extinction learning rates, and a unified emotional learning state parameter. In addition, we quantified response autocorrelation using SCR similarity indices and conducted a statistical comparison of explicit subjective ratings. Below, we detail each of these statistical inference approaches.

Analyses proceeded in three steps. First, we confirmed robust Pavlovian conditioning under sham stimulation and verified that sham performance did not differ between experiments. Second, we tested for amygdala-TUS effects on threat acquisition and subsequent extinction, assessing their temporal specificity across early, mid, and late learning phases. Last, we assessed temporal and target specificity by directly comparing the amygdala-TUS and hippocampus-TUS experiments. We implemented nonparametric cluster-based permutation analyses across all trials to improve sensitivity to temporally specific effects while retaining the same factorial structure and inferential logic as time-binned approaches. This adjustment allowed us to accurately capture transient amygdala-TUS effects during learning. The hierarchical reinforcement learning models were subsequently applied to the same trial-level SCR data to test whether the temporally specific TUS effects identified by the linear mixed-effects models and permutation analyses reflected systematic changes in underlying learning parameters.

The preregistration specified a global four-way interaction model (CS × TUS × TRIAL × EXP), to test whether TUS effects would accumulate gradually across acquisition trials. However, the observed data revealed temporally specific early-phase effects that were not captured by this global model, likely because it averaged across many later trials that showed no evidence of TUS-related modulation.

### Linear mixed-effects models: SCR

We characterized the temporal dynamics of threat and extinction learning using trial-level and time-binned (see the Supplementary Materials, section S7 for details) linear mixed-effects models, implemented using the lme4 package in R (*108*). These models accounted for both within- and between-participant variability by including by-participant random intercepts and random slopes for fixed effects, with random effects structures simplified as necessary to ensure model convergence. Fixed effects included Conditioned Stimulus (threat versus safety), Stimulation (active-TUS versus sham), Trial (as a continuous predictor), and Experiment (amygdala versus hippocampus), as well as their interactions. Time bins were defined as averages within trial bins—early, mid, and late for acquisition; early and late for extinction and re-extinction. The statistical significance of fixed effects was evaluated using type III conditional *F* tests with Kenward-Roger approximation for degrees of freedom via the Anova function in the car package (*109*, *110*). Where significant effects were observed, post hoc pairwise comparisons were conducted using the emmeans package (*111*).

### Nonparametric cluster-based permutation tests

To acknowledge the inherent autocorrelated nature of SCRs over time and to avoid implicitly enforcing assumptions of independence and normality in parametric tests, we adopted nonparametric cluster-based permutation analysis. This allowed us to examine the trial-wise differences in SCR between threat and safety cues across TUS conditions. Cluster-based permutation testing as implemented in the PALM toolbox [version alpha119; (*112*)] was conducted with 10,000 permutations on the SCR data, using a design matrix that included main effects of TUS and CS, as well as their interaction, both within experiments and across experiments for direct comparison. The threshold-free cluster enhancement method was applied to one-dimensional data to enhance sensitivity to contiguous temporal effects.

### Hierarchical reinforcement learning models

Computational modeling was used to infer latent learning mechanisms underlying the observed TUS effects on trial-level SCRs, including learning rates and a unified emotional learning state spanning acquisition and extinction. SCR data from active TUS conditions were analyzed using a set of hierarchical reinforcement learning models implemented in STAN via R (version 4.1.1; RStudio Team, 2021). All models used weakly informative hyperpriors for group-level means and SDs of the free parameters. Specifically, hyperpriors on the group-level means were defined as normal (0,1), and group-level SDs followed a cauchy (0,1) distribution. Individual-level parameters were estimated using noncentered parameterization, with participant-level values drawn from their respective group-level distributions.

Model fitting was performed using four independent Markov chains with 200 warm-up iterations and 1800 post warm-up iterations per chain (8000 total). Posterior estimates for each parameter were extracted and assessed for convergence [Rhat < 1.1; (*113*)].

The acquisition-phase model implemented a canonical Rescorla-Wagner reinforcement learning framework. Trial-wise updates of expected value (EV; probability of shock) for each CS (CUE) were governed by a participant-specific learning rate (LR ∈ [0,1]). On each trial, the estimated outcome probability was updated according to the PE, calculated as the difference between the observed US (0 = unreinforced, 1 = reinforced) and the current EV for the corresponding CUE

$$\text{Prediction error}:{\text{PE}}_{\left[t\right]}={\text{US}}_{\left[t\right]}-{\text{EV}}_{\text{CUE}\left[t\right]}$$

$$\text{RW}\;\text{updating rule}:{\text{EV}}_{\text{CUE}\left[t+1\right]}={\text{EV}}_{\text{CUE}\left[t\right]}\;+\;\text{LR}\;\times \;{\text{PE}}_{\left[t\right]}$$

Observed SCRs were modeled as a linear function of both the current EV and reinforcement history, with additional modulation by participant-specific parameters: conditioned response (CR) intercept and slope for unreinforced trials, combined conditioned and unconditioned response (CR + UR) intercept and slope for reinforced trials, and a residual error term. Model-predicted SCRs were constrained to non-negative values via a soft constraint.

The extinction-phase model was fit to SCR data from the extinction phase, during which no US trials were delivered (i.e., US = 0 for all trials). Participant-specific initial EVs for safety and threat cues were imported from the last trial of the acquisition phase, ensuring that extinction learning was seeded by the individualized outcome of prior conditioning. The model retained the core reinforcement learning architecture, with trial-wise updates governed by a participant-specific LR. Because no reinforcement occurred during extinction, PE was simply the negative of the current EV, and learning progressed through the reduction of previously learned expectations.

The emotional learning state model incorporated observed SCRs across both acquisition and extinction into a unified reinforcement learning framework. Each trial was annotated with a phase indicator (PHASE ∈ {+1 = acquisition, −1 = extinction}). The learning rate, estimated across acquisition and extinction (LR_SHARED_), was dynamically modulated according to both the phase and a participant-specific TUS modulation parameter (TUS), using the formulation

$$\begin{array}{c} {\text{LR}}_{\text{EMOTIONAL}\_\text{STATE}}={\text{LR}}_{\text{SHARED}}\;\times \\ \left(1\;+\;{\text{PHASE}}_{(\text{ACQUISITION},\text{EXTINCTION})\;}\;\times \;\text{TUS}\right) \end{array}$$

This formulation allowed learning to either speed up or slow down, depending on whether the trial occurred during acquisition or extinction, effectively capturing various learning processes across different contexts. It also ensures that learning is centered around LR_SHARED_ and is symmetrically adjusted across phases without introducing negative learning rates.

### SCR similarity index

To investigate the effect of TUS on the acquisition of threat value, we focused on the autocorrelation of SCRs across consecutive trials. The primary analysis involved computing the autocorrelation of SCRs between consecutive trial pairs. Specifically, we computed the lagged correlation between SCR responses on reinforced threat trials (CS+) and subsequent unreinforced threat trials (CS + US). This index quantifies how the SCR to the CS evolves to reflect the value of the US after reinforcement, capturing the degree of postreinforcement cue-value updating. Autocorrelation was calculated separately for two key time lags: from reinforced trial *t*_0_ to unreinforced trial *t*_+1_ (UR-CR similarity) and from unreinforced trial *t*_+1_ to unreinforced trial *t*_+2_ (CR-CR similarity). Paired *t* tests were then conducted to compare the correlation coefficients across experiments (amygdala-TUS versus hippocampus-TUS).

### Subjective ratings

For each experiment, we tested the effect of TUS on subjective postexperiment ratings of retrospective threat probability, arousal, and valence associated with each cue using two-tailed paired-sample *t* tests on the threat > safety differential scores (active versus sham TUS). For placebo-related beliefs and reported sensory experiences associated with TUS, which were not cue specific, we compared the two experiments on the proportion of participants reporting tactile, thermal, or auditory sensations, believing that the stimulation was real versus placebo, using chi-square tests of independence (Fisher’s exact test where appropriate).

## Supplementary Materials

### This PDF file includes:

Supplementary Text Figs. S1 to S3 Table S1 References
