# Supplementary material for: The human amygdala in threat learning and extinction
Source: Sci Adv. 2026 Mar 25;12(13):eaea8233. doi: 10.1126/sciadv.aea8233 (PMC13015908; doi:10.1126/sciadv.aea8233)
Supplement: Supplementary file 1 — Supplementary Text Figs. S1 to S3 Table S1 References [file sciadv.aea8233_sm.pdf]

Science Advances
AAAS

Supplementary Materials for

The human amygdala in threat learning and extinction

Sjoerd Meijer *et al.*

Corresponding author: Sjoerd Meijer, sjoerd.meijer@donders.ru.nl

Sci. Adv. **12**, eaea8233 (2026)
DOI: 10.1126/sciadv.aea8233

This PDF file includes:

Supplementary Text
Figs. S1 to S3
Table S1
References

Supplementary Text

Section S1. Pavlovian conditioning effects in the baseline sham condition (Experiment I)

Successful differential threat conditioning was observed in the baseline sham condition of the amygdala-TUS experiment, with individuals showing stronger conditioned responses to the unreinforced threat (CS+) compared to safety cue (CS–) ($F_{(1, 173)} = 48.87$, $p < 0.0001$, $\eta_p^2 = 0.22$; **fig. 1B**). When the temporal delay between conditioned stimulus (CS) presentation and shock (US) administration is short, skin-conductance responses (SCRs) can reflect a mixture of conditioned and unconditioned responding. Consistent with this, the strongest SCRs were observed for reinforced threat trials (CS+US) ($F_{(1, 24)} = 110.86$, $p < 0.0001$, $\eta_p^2 = 0.82$).

Participants also demonstrated explicit threat learning as indexed by post-experiment ratings of retrospective threat probability (threat > safety; $F_{(1, 24)} = 241.15$, $p < 0.0001$, $\eta_p^2 = 0.91$), negative valence ($F_{(1, 48)} = 38.45$, $p < 0.0001$, $\eta_p^2 = 0.44$), and heightened arousal ($F_{(1, 48)} = 27.95$, $p < 0.0001$, $\eta_p^2 = 0.37$).

Threat acquisition

Participants rapidly acquired conditioned threat responses, as revealed by a significant threat > safety cluster beginning early during acquisition and continuing until the end of acquisition (acquisition cluster: trials 3 – 15, $p_{\text{fwe}} < 0.0001$; $F_{(1, 1073)} = 19.91$, $p < 0.0001$, $\eta_p^2 = 0.02$; **fig. 1C**). Time-binned analyses show robust differential conditioned responses building up from the early period toward the middle and late periods of acquisition (early: $F_{(1, 24)} = 14.94$, $p = 0.0007$, $\eta_p^2 = 0.38$; mid: $F_{(1, 24)} = 35.54$, $p < 0.0001$, $\eta_p^2 = 0.60$; late: $F_{(1, 24)} = 33.74$, $p < 0.0001$, $\eta_p^2 = 0.58$; **fig. S1A**), suggesting that the early acquisition trials are critical for the rapid formation and subsequent expression of the threat memory trace (*115*).

Threat retention

To probe threat memory expression and subsequent extinction learning processes, threat cues are no longer reinforced after the acquisition phase (*47*). Participants demonstrate retention of conditioned responding when confronted with the threat following a short temporal delay introduced by a distractor task, exhibiting conditioned responses similar to those observed at the end of acquisition (RETENT * CS interaction: $p > 0.05$; CS main effect: $F_{(1, 24)} = 10.28$, $p = 0.0038$, $\eta_p^2 = 0.30$; **fig. S1B**).

Extinction

In the following extinction trials, participants require several trials before conditioned threat responses extinguish (extinction cluster: trials 1 – 6, $p_{\text{fwe}} < 0.0001$; $F_{(1, 66)} = 3.05$, $p = 0.0856$, $\eta_p^2 = 0.04$; **fig. 1D**), reaching levels comparable to the safety cue by late extinction (early: $F_{(1, 24)} = 9.50$, $p = 0.0051$, $\eta_p^2 = 0.28$; late: $F_{(1, 24)} = 1.27$, $p = 0.2711$; **fig. S1C**).

Recovery following reinstatement

To determine whether the original threat memory trace persists following extinction, three uncued US presentations are delivered to reactivate the threat-reinforcer association (*47*). This led to recovery of conditioned threat responding, comparing the end of extinction to the first trial after reinstatement (REINST * CS interaction: $F_{(1, 48)} = 6.57$, $p = 0.0136$, $\eta_p^2 = 0.12$; **fig. S1D**). These

findings support the view that extinction does not erase the original threat memory but rather leads to inhibition or updating (*79*, *114*).

Re-extinction

After recovery following reinstatement, participants show an immediate decline in conditioned responding ($F_{(1,\ 188)} = 0.21$, $p = 0.6475$; **fig. 1G/S1E**), underscoring the potency of the initial extinction process.

Section S2. Amygdala-TUS does not impact reinforced threat responses or habituation

To confirm that amygdala-TUS effects on early conditioning are specific to associative learning during acquisition and not driven by non-associative processes (*47*), we examined alternative explanations, including modulation of reinforced threat responses (CS+US) or habituation. First, responses on reinforced trials did not differ between amygdala-TUS and sham conditions (p = 0.7649), suggesting that amygdala-TUS did not modulate reinforced threat responses. We note that given the short temporal delay between CS onset and US delivery in our paradigm, these responses likely reflect a mixture of conditioned (anticipatory) and unconditioned (shock-evoked) components rather than isolated US responses. Second, accounting for logarithmic habituation dynamics significantly improved model fit (model comparison: $\chi^2 = 229$, $p < 0.0001$, ΔAIC = -277, ΔBIC = -221; habituation model: $b_{\text{CS:TUS:TRIAL}} = -0.0112$, $F_{(1,\ 48)} = 5.09$, $p = 0.0287$, $\eta_p^2 = 0.10$), indicating that TUS effects are not attributable to changes in habituation. These findings confirm that amygdala-TUS specifically disrupts acquisition of conditioned threat responding rather than indirectly affecting threat learning via changes in non-associative processes.

Section S3. Subjective arousal and valence scores

In the amygdala-TUS experiment, threat as compared to safety cues were rated as more negative in valence in post-experiment ratings under both active TUS ($t_{(24)} = 4.95$, $p < 0.0001$) and sham stimulation ($t_{(24)} = 5.66$, $p < 0.0001$). Similarly, arousal ratings were higher for threat than safety cues under both active TUS ($t_{(24)} = 3.08$, $p = 0.0052$) and sham stimulation ($t_{(24)} = 5.15$, $p < 0.0001$).

The hippocampus-TUS experiment showed the same pattern for both valence (active TUS: $t_{(24)} = 6.36$, $p < 0.0001$; sham: $t_{(24)} = 7.59$, $p < 0.0001$) and arousal (active TUS: $t_{(24)} = 5.94$, $p < 0.0001$; sham: $t_{(24)} = 4.27$, $p = 0.0003$).

Section S4. Amygdala-TUS effects are target-specific

As opposed to the amygdala-TUS experiment, we found no effects of hippocampus-TUS compared to sham on differential conditioned responding during threat acquisition or extinction (all p-values > 0.05; **fig. S2A$_{\text{a-b}}$; S2B$_{\text{a-b}}$**), confirming the specificity of amygdala-TUS effects on associative learning. Additionally, no other differences were observed between hippocampus-TUS and sham conditions (all p-values > 0.05; **fig. S2C; S2E**), indicating that our cued delay conditioning paradigm in which the US is presented simultaneously with the CS does not introduce strong posterior hippocampal-dependencies typical of contextual conditioning studies (*40–43*, *45*).

Section S5. Shared TUS confound on threat retention across experiments

The comparison of active-TUS *vs.* sham conditions across experiments revealed a target non-specific effect, where a (marginal) main effect of TUS on threat retention was observed in both amygdala-TUS and hippocampus-TUS experiments with stronger conditioned responding both at

the end of acquisition as well as during the first trial post-acquisition (Experiment I: $F_{(1, 24)} = 4.00$, $p = 0.0581$, $\eta_p^2 = 0.14$, **fig. S1D**; Experiment II: $F_{(1, 58)} = 6.61$, $p = 0.0127$, $\eta_p^2 = 0.10$, **fig. S2D**). We speculate somatosensory percepts of TUS contribute to multi-modal cueing (*73*), building up across longer periods of online TUS, particularly once the threat association is learned and attention may shift away from ongoing associative learning. These findings highlight the importance of using an active control site to interpret TUS effects with high specificity.

Subjective reports of peripheral sensations and placebo-related beliefs associated with TUS did not differ between the active (amygdala) and active-control (hippocampus) experiments (Experiment I *vs.* II; all *p*-values > 0.05). In Experiment I (N = 25), auditory sensations were reported by 25/25 participants, tactile by 21/25, thermal by 4/25, and 21/25 believed they received real stimulation. In Experiment II (N = 25), auditory sensations were reported by 25/25 participants, tactile by 19/25, thermal by 3/25, and 22/25 believed they received real stimulation. Overall, participants' subjective experiences of TUS were comparable across experiments.

Section S6. Amygdala-TUS impacts threat-value updating but not maintenance

A key question is how the amygdala drives threat learning, specifically how the CS acquires the value of the US to trigger threat responses, leading to conditioned behavior. Animal models suggest that threat learning involves the neural representation of the CS becoming more similar to that of the US, with the basolateral amygdala serving as a convergence site for both signals (*54*). We hypothesized that amygdala-TUS would selectively interfere with threat-value updating, rather than affecting the maintenance of threat-value across unreinforced trials during acquisition. To test this, we quantify UR-CR similarity as the lagged autocorrelation of SCRs between consecutive reinforced (CS+US) and unreinforced threat (CS+) trials (see *Materials and Methods*). This index measures post-reinforcement cue-value updating, assuming that as neural representations of the US and CS converge, so too does their behavioral expression. Consistent with our hypothesis, amygdala-TUS reduced UR-CR similarity compared to hippocampus-TUS ($t_{(43)} = -2.3$, $p = 0.0256$, *Cohen's* $d_{(pooled\ SD)} = -0.65$; **fig. S3**), indicating a disruption in how the CS acquires US value—a critical mechanism in initial threat learning (*53*). This effect was specific to post-reinforcement value updating and did not generalize to threat-value maintenance: amygdala-TUS did not affect CR-CR similarity (SCR responses on consecutive unreinforced threat trials; $t_{(47)} = 0.42$, $p = 0.6789$; **fig. S3**). These findings suggest that amygdala-TUS selectively modulates value updating following reinforcement, potentially due to a diminished influence of reinforcement-related prediction errors on updating of expected value, which is modulated by an individuals' learning rate.

Section S7. Cue-specific trial numbering and time bins.

Consistent with previous human threat conditioning research investigating temporal learning dynamics (*47*), we numbered trials separately for each CS to capture cue-specific learning trajectories. During acquisition, analyses of the threat cue (CS+) were restricted to unreinforced trials (15 of 30 total CS+ trials). To align the safety condition (30 trials per cue) with these temporal dynamics, we averaged responses over adjacent safety-trial pairs, yielding 15 matched data points while retaining all unreinforced trials. During extinction—when no shocks occur—each cue presentation is similarly numbered in sequence.

To parallel previous human threat conditioning studies, we also grouped trials into time bins. For acquisition, trials were divided into three bins—early (trials 1–5), middle (6–10), and late (11–15). Extinction and re-extinction trials were split into two bins—early (2–5) and late (6–10)—to capture key changes in conditioned threat responding over time while maintaining adequate trial numbers per bin. The first post-acquisition and post-reinstatement trials were used in the threat retention and recovery following reinstatement analyses, respectively.

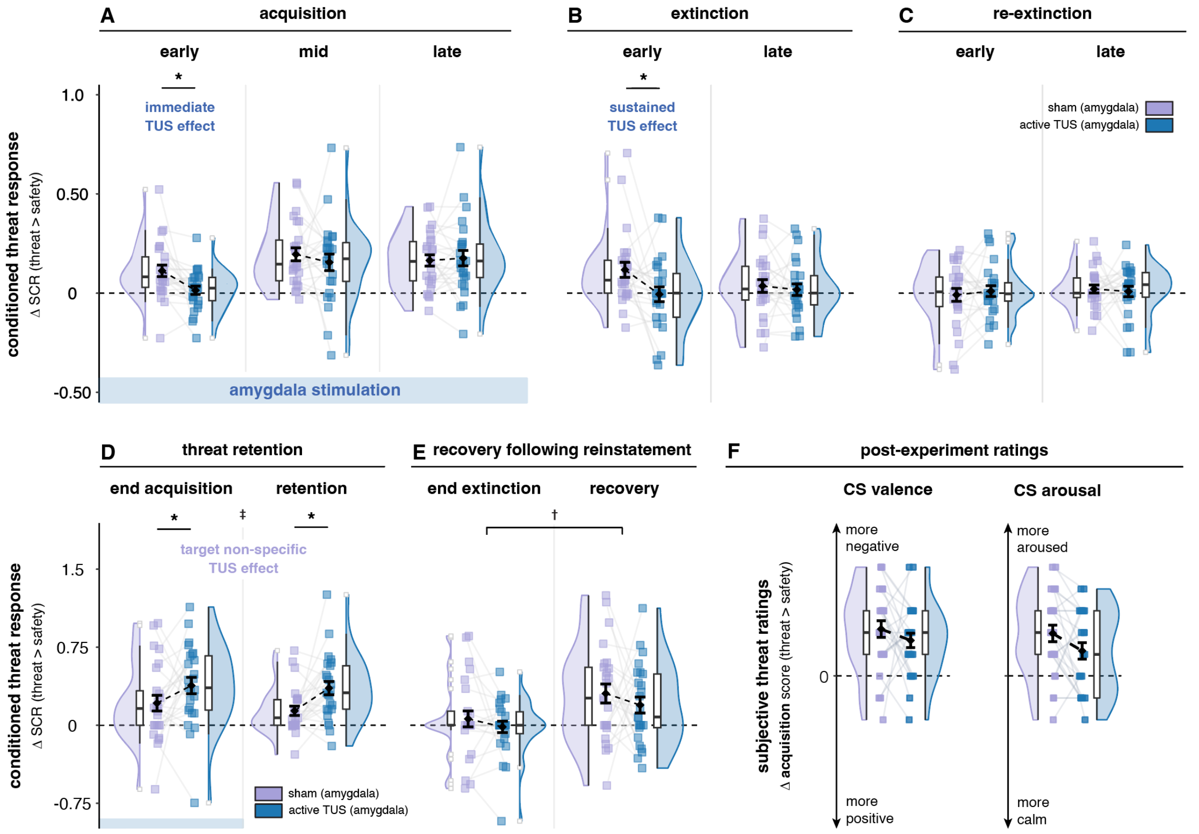

Fig. S1. Experiment I: Amygdala-TUS selectively impairs early threat learning and enhances subsequent extinction*. *(A–E) Time-binned differential SCRs (threat > safety) across Pavlovian conditioning phases*. *(A) Acquisition*: *Amygdala-TUS interferes with early threat learning;* ***(B) Extinction: *Amygdala-TUS augments early extinction.* ***(C) Re-extinction***: *Amygdala-TUS shows no significant effects on re-extinction.* ***(D) Threat retention***: *‡ marks main effect of TUS;* ***(E) Recovery following reinstatement***: *† marks main effect of reinstatement;* ***(F) Post-experiment subjective ratings (CS+ > CS–)***: *Participants rated threat cues as more negative in valence and more arousing than safety cues, consistent with successful threat acquisition. These subjective post-experiment ratings were unaffected by amygdala-TUS. Boxplots and rainclouds illustrate distribution and density of individual responses. Asterisks (*) indicate significant TUS × CS interactions ($p < 0.05$). These results highlight selective modulation of conditioned responding during threat acquisition and subsequent extinction by amygdala-TUS.*

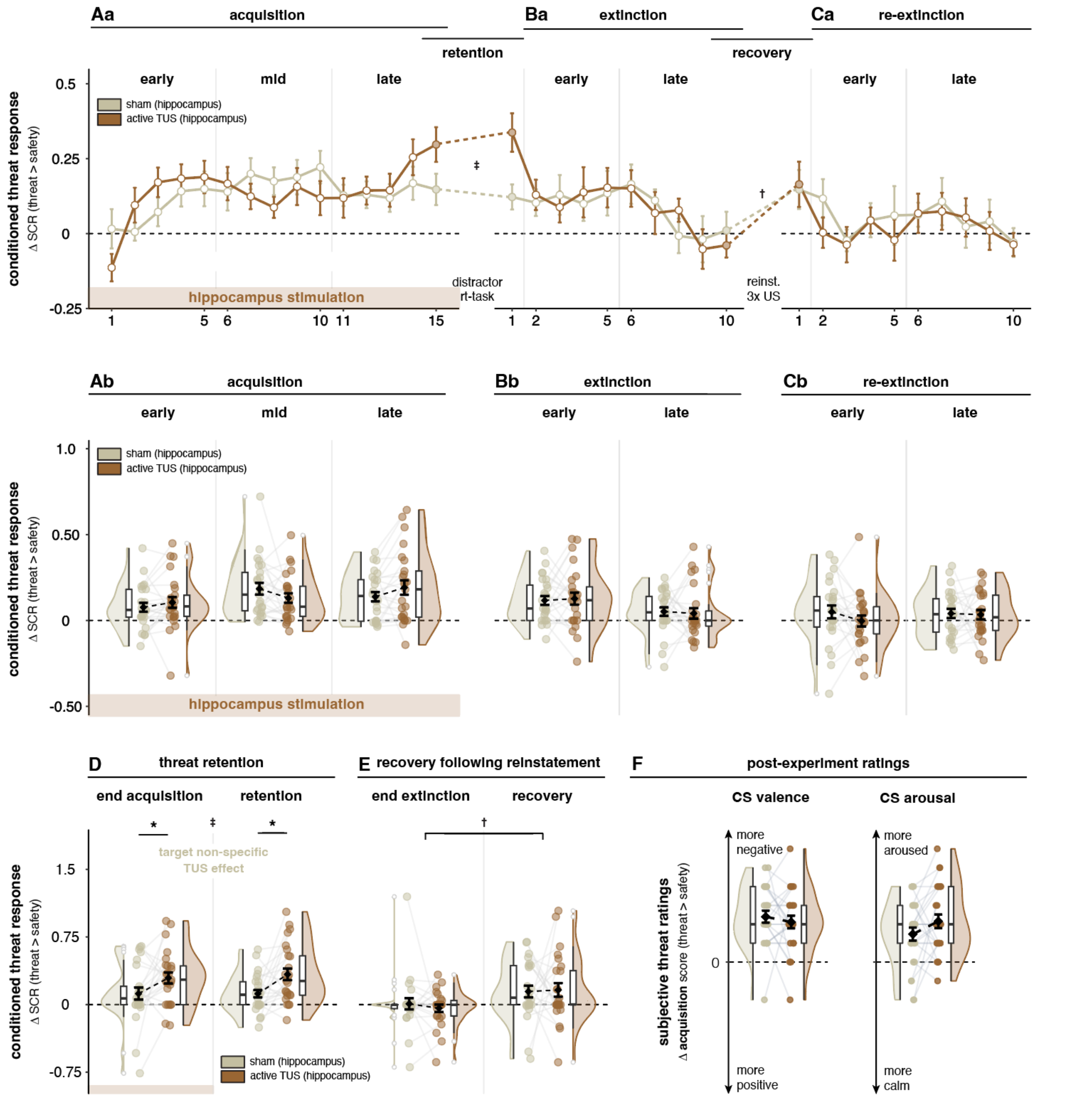

Fig. S2. Experiment II: Hippocampus-TUS does not influence threat learning or extinction, confirming amygdala specificity of TUS effects*. *(A–C)* *Differential SCRs (threat > safety) during acquisition, extinction, and re-extinction under hippocampus-TUS compared to sham stimulation.* ***(A) Acquisition*: Temporal dynamics (A_a) and time-binned data (A_b) show no significant hippocampus-TUS effects on threat acquisition (all $p > 0.05$).* ***(B) Extinction****: Temporal dynamics (B_a) and time-binned data (B_b) confirm hippocampus-TUS does not affect conditioned responding during extinction.* ***(C) Re-extinction****: Hippocampus-TUS had no impact on conditioned responding during re-extinction.* ***(D) Threat retention*** *and* ***(E) Recovery following reinstatement****: hippocampus-specific TUS showed no effects on conditioned responding during threat retention or recovery following reinstatement. A target non-specific TUS effect on threat retention (‡ main TUS effect) occurred similarly in both hippocampus- and amygdala-TUS experiments, reflecting*

potential somatosensory cueing during TUS application. † Indicates a main reinstatement effect (CS × reinstatement interaction). ***(F) Post-experiment subjective ratings (CS+ > CS–)****: Participants rated threat cues as more negative in valence and more arousing than safety cues, consistent with successful threat acquisition. These post-experiment ratings, made with reference to the acquisition phase, were unaffected by hippocampus-TUS. Boxplots and raincloud plots display individual response distributions and densities. These findings demonstrate the anatomical specificity of amygdala-TUS effects on threat acquisition and extinction and underline the necessity of using active-control targets to control for peripheral sensory and placebo effects inherent in TUS application.*

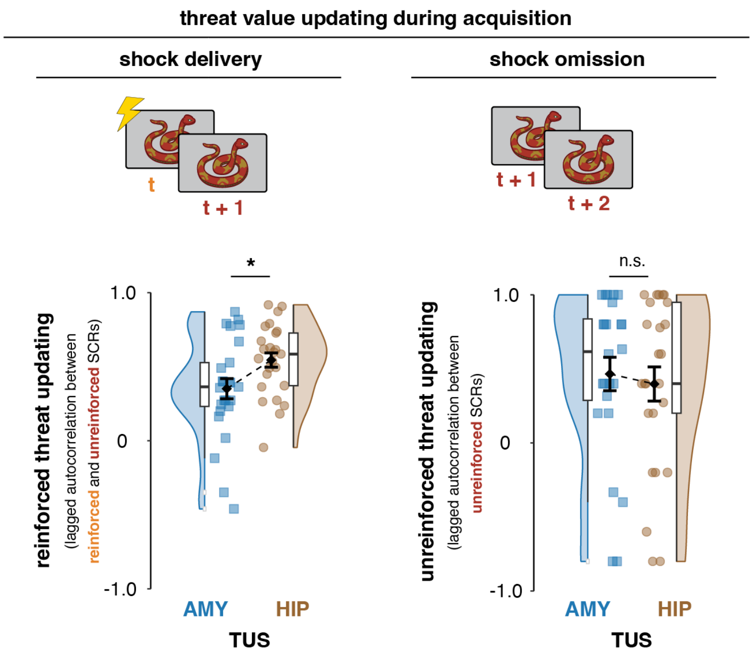

***Fig. S3. Amygdala-TUS selectively disrupts threat-value updating after reinforcement*.**
Left: *Threat-value updating following reinforcement. Individual participant data showing UR–CR similarity (lagged autocorrelation of SCRs between reinforced [threat+shock] and subsequent unreinforced [threat] trials) for participants receiving active amygdala-TUS (blue; N = 25) or hippocampus-TUS (brown; N = 25). Group means are indicated by black diamonds (± SEM). Boxplots and raincloud plots illustrate data distribution and density. Amygdala-TUS significantly reduced UR–CR similarity compared to hippocampus-TUS, indicating impaired updating of threat values following reinforcement ($t_{(43)} = -2.31, p = 0.0256$).* ***Right***: *Maintenance of acquired threat-value. CR–CR similarity (lagged autocorrelation between consecutive unreinforced threat trials) comparing amygdala-TUS and hippocampus-TUS groups. There was no significant difference between groups ($p = 0.6789$), indicating that amygdala-TUS did not affect threat-value maintenance across trials. These results suggest that amygdala neuromodulation selectively interferes with reinforcement-driven threat updating rather than general threat-value stability.*

Table S1: TUS specifications per ITRUSST standardized reporting guidelines.

Transducer and drive system parameters

Transducer	Centre frequency	Radius of curvature	Aperture diameter	Number of elements	Element distribution	Matching network	Drive systems
250-4CH*	250 kHz	64 mm	64 mm	4	Annular array, bowl, equal area	4-channel electrical impedance matching network	TPO-105-010<br>TPO-203-035

**Manufacturer: Sonic Concepts Inc., Bothell, WA; Supplier/support: BrainBox Ltd., Cardiff, UK.*

Driving system settings

Transducer	Operating frequency	TPO output I_{sppa} setting	Focal depth setting
250-4CH	250 kHz	40 W/cm^2	61.5 mm

Pulse timing parameters

	Duration	Ramp duration	Ramp shape	Repetition interval (frequency)
**Pulse**	90 ms	30 ms	Tukey	200 ms (5 Hz)
**Pulse Train**	1 s			

Free field pressure parameters

Transducer	I_{sppa}	Axial position of I_{sppa}*	Focus -3 dB	Focus -6 dB
250-4CH	38.43 W/cm^2	61.5 mm	Volume: 48.6 mm^3	Volume: 75.8 mm^3

**Relative to the exit plane of the transducer.*

Safety metrics

TUS target	$I_{sppa\_TC\_SIM}$[1]	$MI_{\_TC\_SIM}$[2]	$MI_{\_TC\_CONS}$[3]	Max. TR[4]	CEM43 °C[5]
amygdala	12.3 W/cm^2	1.22	1.54	1.09 °C	<0.1
hippocampus	15.5 W/cm^2	1.06	1.54	0.73 °C	<0.1

[1]*Representative simulated transcranial I_{sppa} in the brain.*

[2]*Representative simulated transcranial mechanical index (MI_{TC_SIM}) in the brain.*

[3]*Conservative estimate of transcranial mechanical index (MI_{TC_CONS}) using the open-source TUS calculator*. Derating values: Diameter of transducer: 64 mm; Focal depth: 61.5 mm; Skull thickness: 10 mm (worst-case assumption of thickness of skull); Scalp thickness: 5.8 mm; Coupling thickness: 5 mm; US beam diameter on skin: 58.8 mm; US beam diameter on skull: 52.8 mm; Skull pressure transmission: 72% (116); Brain pressure transmission: 94%; Attenuation coefficient: 0.5 dB/cm/MHz.*

[4]*Maximum simulated thermal rise (TR).*

[5]*Thermal dose in cumulative equivalent minutes (CEM) at 43 °C.*

*https://www.socsci.ru.nl/fusinitiative/tuscalculator/

REFERENCES

1. D. Pare, S. Duvarci, Amygdala microcircuits mediating fear expression and extinction. *Curr. Opin. Neurobiol.* **22**, 717–723 (2012).

2. P. Tovote, J. P. Fadok, A. Lüthi, Neuronal circuits for fear and anxiety. *Nat. Rev. Neurosci.* **16**, 317–331 (2015).

3. M. G. Craske, D. Hermans, B. Vervliet, State-of-the-art and future directions for extinction as a translational model for fear and anxiety. *Philos. Trans. R. Soc. Lond. B Biol. Sci.* **373**, 20170025 (2018).

4. P. Duits, D. C. Cath, S. Lissek, J. J. Hox, A. O. Hamm, I. M. Engelhard, M. A. van den Hout, J. M. P. Baas, Updated meta-analysis of classical fear conditioning in the anxiety disorders. *Depress. Anxiety* **32**, 239–253 (2015).

5. M. B. L. Careaga, C. E. N. Girardi, D. Suchecki, Understanding posttraumatic stress disorder through fear conditioning, extinction and reconsolidation. *Neurosci. Biobehav. Rev.* **71**, 48–57 (2016).

6. T. Beckers, D. Hermans, I. Lange, L. Luyten, S. Scheveneels, B. Vervliet, Understanding clinical fear and anxiety through the lens of human fear conditioning. *Nat. Rev. Psychol.* **2**, 233–245 (2023).

7. F. M. Kausche, H. P. Carsten, K. M. Sobania, A. Riesel, Fear and safety learning in anxiety- and stress-related disorders: An updated meta-analysis. *Neurosci. Biobehav. Rev.* **169**, 105983 (2025).

8. J. Liu, M. S. Totty, H. Bayer, S. Maren, Integrating aversive memories in the basolateral amygdala. *Biol. Psychiatry* **98**, 746–755 (2025).

9. M. Davis, The role of the amygdala in fear and anxiety. *Annu. Rev. Neurosci.* **15**, 353–375 (1992).

10. J. E. LeDoux, Emotion circuits in the brain. *Annu. Rev. Neurosci.* **23**, 155–184 (2000).

11. S. Maren, Neurobiology of pavlovian fear conditioning. *Annu. Rev. Neurosci.* **24**, 897–931 (2001).

12. E. A. Phelps, J. E. LeDoux, Contributions of the amygdala to emotion processing: From animal models to human behavior. *Neuron* **48**, 175–187 (2005).

13. M. J. D. Miserendino, C. B. Sananes, K. R. Melia, M. Davis, Blocking of acquisition but not expression of conditioned fear-potentiated startle by NMDA antagonists in the amygdala. *Nature* **345**, 716–718 (1990).

14. M. T. Rogan, U. V. Stäubli, J. E. LeDoux, Fear conditioning induces associative long-term potentiation in the amygdala. *Nature* **390**, 604–607 (1997).

15. K. Nader, G. E. Schafe, J. E. Le Doux, Fear memories require protein synthesis in the amygdala for reconsolidation after retrieval. *Nature* **406**, 722–726 (2000).

16. J.-H. Han, S. A. Kushner, A. P. Yiu, H.-L. Hsiang, T. Buch, A. Waisman, B. Bontempi, R. L. Neve, P. W. Frankland, S. A. Josselyn, Selective erasure of a fear memory. *Science* **323**, 1492–1496 (2009).

17. B. F. Grewe, J. Gründemann, L. J. Kitch, J. A. Lecoq, J. G. Parker, J. D. Marshall, M. C. Larkin, P. E. Jercog, F. Grenier, J. Z. Li, A. Lüthi, M. J. Schnitzer, Neural ensemble dynamics underlying a long-term associative memory. *Nature* **543**, 670–675 (2017).

18. R. M. Visser, J. Bathelt, H. S. Scholte, M. Kindt, Robust BOLD responses to faces but not to conditioned threat: Challenging the amygdala’s reputation in human fear and extinction learning. *J. Neurosci.* **41**, 10278–10292 (2021).

19. Z. Wen, C. M. Raio, E. F. Pace-Schott, S. W. Lazar, J. E. LeDoux, E. A. Phelps, M. R. Milad, Temporally and anatomically specific contributions of the human amygdala to threat and safety learning. *Proc. Natl. Acad. Sci. U.S.A.* **119**, e2204066119 (2022).

20. J. Radua, H. S. Savage, E. Vilajosana, A. Jamieson, B. Abler, F. Åhs, T. Beckers, N. Cardoner, J. M. Cisler, J. B. Diniz, D. R. Bach, S. Elsenbruch, S. G. Greening, D. J. Holt, A. N.

Kaczkurkin, A. Keil, M. Kindt, K. Koch, K. S. LaBar, C. L. Lam, C. L. Larson, T. B. Lonsdorf, C. J. Merz, K. A. McLaughlin, Y. Neria, D. S. Pine, C. M. van Reekum, A. J. Shackman, C. Soriano-Mas, V. I. Spoormaker, D. M. Stout, B. Straube, T. Straube, L. Tuominen, R. M. Visser, L. Ahumada, V. Arolt, M. C. Batistuzzo, P. R. Bazán, E. E. Biggs, M. Cano, P. Chavarría-Elizondo, S. E. Cooper, U. Dannlowski, V. de la Peña-Arteaga, S. N. DeCross, K. Domschke, M. R. Ehlers, J. L. Graner, A. O. Hamm, M. J. Herrmann, A. A. Huggins, A. Icenhour, A. Juaneda-Seguí, M. Junghoefer, T. Kircher, K. Koelkebeck, M. Kuhn, F. Labrenz, S. M. Lissek, M. Lotze, U. Lueken, J. Margraf, I. Martínez-Zalacaín, R. Moeck, J. Morriss, M. Ortuño, A. Pittig, D. Porta-Casteras, J. Richter, I. C. Ridderbusch, W. Rief, K. Roesmann, J. Rosén, A. N. Rußmann, R. Sjouwerman, J. Spohrs, A. Ströhle, B. Suarez-Jimenez, M. Ulrich, H.-U. Wittchen, X. Zhu, L. Waller, H. Walter, P. M. Thompson, J. M. Bas-Hoogendam, N. A. Groenewold, D. J. Stein, N. J. Van der Wee, J. E. Dunsmoor, A. F. Marquand, B. J. Harrison, M. A. Fullana, Neural correlates of human fear conditioning and sources of variability in 2199 individuals. *Nat. Commun.* **16**, 7869 (2025).

21. C. Büchel, J. Morris, R. J. Dolan, K. J. Friston, Brain systems mediating aversive conditioning: An event-related fMRI study. *Neuron* **20**, 947–957 (1998).

22. K. S. LaBar, J. C. Gatenby, J. C. Gore, J. E. LeDoux, E. A. Phelps, Human amygdala activation during conditioned fear acquisition and extinction: A mixed-trial fMRI study. *Neuron* **20**, 937–945 (1998).

23. D. C. Knight, C. N. Smith, D. T. Cheng, E. A. Stein, F. J. Helmstetter, Amygdala and hippocampal activity during acquisition and extinction of human fear conditioning. *Cogn. Affect. Behav. Neurosci.* **4**, 317–325 (2004).

24. E. A. Phelps, M. R. Delgado, K. I. Nearing, J. E. LeDoux, Extinction learning in humans: Role of the amygdala and vmPFC. *Neuron* **43**, 897–905 (2004).

25. M.-L. Mechias, A. Etkin, R. Kalisch, A meta-analysis of instructed fear studies: Implications for conscious appraisal of threat. *Neuroimage* **49**, 1760–1768 (2010).

26. D. R. Bach, N. Weiskopf, R. J. Dolan, A stable sparse fear memory trace in human amygdala. *J. Neurosci.* **31**, 9383–9389 (2011).

27. M. A. Fullana, B. J. Harrison, C. Soriano-Mas, B. Vervliet, N. Cardoner, A. Àvila-Parcet, J. Radua, Neural signatures of human fear conditioning: An updated and extended meta-analysis of fMRI studies. *Mol. Psychiatry* **21**, 500–508 (2016).

28. A. Bechara, D. Tranel, H. Damasio, R. Adolphs, C. Rockland, A. Damasio, Double dissociation of conditioning and declarative knowledge relative to the amygdala and hippocampus in humans. *Science* **269**, 1115–1118 (1995).

29. E. A. Phelps, K. S. Labar, A. K. Anderson, K. J. O'connor, R. K. Fulbright, D. D. Spencer, Specifying the contributions of the human amygdala to emotional memory: A case study. *Neurocase* **4**, 527–540 (1998).

30. F. Klumpers, B. Morgan, D. Terburg, D. J. Stein, J. van Honk, Impaired acquisition of classically conditioned fear-potentiated startle reflexes in humans with focal bilateral basolateral amygdala damage. *Soc. Cogn. Affect. Neurosci.* **10**, 1161–1168 (2015).

31. T. Chou, T. Deckersbach, B. Guerin, K. Sretavan Wong, B. M. Borron, A. Kanabar, A. N. Hayden, M. P. Long, M. Daneshzand, E. F. Pace-Schott, D. D. Dougherty, Transcranial focused ultrasound of the amygdala modulates fear network activation and connectivity. *Brain Stimul.* **17**, 312–320 (2024).

32. B. Hoang-Dang, S. E. Halavi, N. M. Rotstein, N. M. Spivak, N. H. Dang, L. Cvijanovic, S. H. Hiller, M. Vallejo-Martelo, B. M. Rosenberg, A. Swenson, S. Becerra, M. Sun, M. E. Revett, D. Kronemyer, R. Berlow, M. G. Craske, N. Suthana, M. M. Monti, T. D. Zbozinek, S. Y. Bookheimer, T. P. Kuhn, Transcranial focused ultrasound targeting the amygdala may increase psychophysiological and subjective negative emotional reactivity in healthy older adults. *Biol. Psychiatry Glob. Open Sci.* **4**, 100342 (2024).

33. B. R. Barksdale, L. Enten, A. DeMarco, R. Kline, M. K. Doss, C. B. Nemeroff, G. A. Fonzo, Low-intensity transcranial focused ultrasound amygdala neuromodulation: A double-blind sham-

controlled target engagement study and unblinded single-arm clinical trial. *Mol. Psychiatry* **30**, 4497–4511 (2025).

34. D. Folloni, L. Verhagen, R. B. Mars, E. Fouragnan, C. Constans, J.-F. Aubry, M. F. S. Rushworth, J. Sallet, Manipulation of subcortical and deep cortical activity in the primate brain using transcranial focused ultrasound stimulation. *Neuron* **101**, 1109–1116.e5 (2019).

35. S. N. Yaakub, T. A. White, J. Roberts, E. Martin, L. Verhagen, C. J. Stagg, S. Hall, E. F. Fouragnan, Transcranial focused ultrasound-mediated neurochemical and functional connectivity changes in deep cortical regions in humans. *Nat. Commun.* **14**, 5318 (2023).

36. E. Martin, M. Roberts, I. F. Grigoras, O. Wright, T. Nandi, S. W. Rieger, J. Campbell, T. den Boer, B. T. Cox, C. J. Stagg, B. E. Treeby, Ultrasound system for precise neuromodulation of human deep brain circuits. *Nat. Commun.* **16**, 8024 (2025).

37. T. S. Riis, D. A. Feldman, A. J. Losser, A. Okifuji, J. Kubanek, Noninvasive targeted modulation of pain circuits with focused ultrasonic waves. *Pain* **165**, 2829 (2024), 2839.

38. R. A. Rescorla, A. R. Wagner, "A theory of Pavlovian conditioning: Variations in the effectiveness of reinforcement and nonreinforcement" in *Classical Conditioning II: Current Research and Theory*, A. H. Black, W. F. Prokasy, Eds. (Appleton Century Crofts, 1972), pp. 64–99.

39. R. Abend, D. Burk, S. G. Ruiz, A. L. Gold, J. L. Napoli, J. C. Britton, K. J. Michalska, T. Shechner, A. M. Winkler, E. Leibenluft, D. S. Pine, B. B. Averbeck, Computational modeling of threat learning reveals links with anxiety and neuroanatomy in humans. *eLife* **11**, e66169 (2022).

40. J. J. Kim, M. S. Fanselow, Modality-specific retrograde amnesia of fear. *Science* **256**, 675–677 (1992).

41. R. G. Phillips, J. E. LeDoux, Differential contribution of amygdala and hippocampus to cued and contextual fear conditioning. *Behav. Neurosci.* **106**, 274–285 (1992).

42. S. Maren, G. Aharonov, M. S. Fanselow, Neurotoxic lesions of the dorsal hippocampus and Pavlovian fear conditioning in rats. *Behav. Brain Res.* **88**, 261–274 (1997).

43. S. G. Anagnostaras, S. Maren, M. S. Fanselow, Temporally graded retrograde amnesia of contextual fear after hippocampal damage in rats: Within-subjects examination. *J. Neurosci.* **19**, 1106–1114 (1999).

44. D. C. Knight, D. T. Cheng, C. N. Smith, E. A. Stein, F. J. Helmstetter, Neural substrates mediating human delay and trace fear conditioning. *J. Neurosci.* **24**, 218–228 (2004).

45. A. Marschner, R. Kalisch, B. Vervliet, D. Vansteenwegen, C. Büchel, Dissociable roles for the hippocampus and the amygdala in human cued versus context fear conditioning. *J. Neurosci.* **28**, 9030–9036 (2008).

46. R. Polanía, M. A. Nitsche, C. C. Ruff, Studying and modifying brain function with non-invasive brain stimulation. *Nat. Neurosci.* **21**, 174–187 (2018).

47. T. B. Lonsdorf, M. M. Menz, M. Andreatta, M. A. Fullana, A. Golkar, J. Haaker, I. Heitland, A. Hermann, M. Kuhn, O. Kruse, S. Meir Drexler, A. Meulders, F. Nees, A. Pittig, J. Richter, S. Römer, Y. Shiban, A. Schmitz, B. Straube, B. Vervliet, J. Wendt, J. M. P. Baas, C. J. Merz, Don't fear 'fear conditioning': Methodological considerations for the design and analysis of studies on human fear acquisition, extinction, and return of fear. *Neurosci. Biobehav. Rev.* **77**, 247–285 (2017).

48. L. Weiskrantz, Behavioral changes associated with ablation of the amygdaloid complex in monkeys. *J. Comp. Physiol. Psychol.* **49**, 381–391 (1956).

49. E. A. Antoniadis, J. T. Winslow, M. Davis, D. G. Amaral, Role of the primate amygdala in fear-potentiated startle: Effects of chronic lesions in the rhesus monkey. *J. Neurosci.* **27**, 7386–7396 (2007).

50. E. A. Antoniadis, J. T. Winslow, M. Davis, D. G. Amaral, The non-human primate amygdala is necessary for the acquisition but not the retention of fear-potentiated startle. *Biol. Psychiatry* **65**, 241–248 (2009).

51. A. M. Poulos, V. Li, S. S. Sterlace, F. Tokushige, R. Ponnusamy, M. S. Fanselow, Persistence of fear memory across time requires the basolateral amygdala complex. *Proc. Natl. Acad. Sci. U.S.A.* **106**, 11737–11741 (2009).

52. A. M. Kazama, E. Heuer, M. Davis, J. Bachevalier, Effects of neonatal amygdala lesions on fear learning, conditioned inhibition, and extinction in adult macaques. *Behav. Neurosci.* **126**, 392–403 (2012).

53. G. J. Quirk, J. C. Repa, J. E. LeDoux, Fear conditioning enhances short-latency auditory responses of lateral amygdala neurons: Parallel recordings in the freely behaving rat. *Neuron* **15**, 1029–1039 (1995).

54. J. P. Johansen, H. Hamanaka, M. H. Monfils, R. Behnia, K. Deisseroth, H. T. Blair, J. E. LeDoux, Optical activation of lateral amygdala pyramidal cells instructs associative fear learning. *Proc. Natl. Acad. Sci. U.S.A.* **107**, 12692–12697 (2010).

55. W. O. Jenkins, J. C. Stanley Jr., Partial reinforcement: A review and critique. *Psychol. Bull.* **47**, 193–234 (1950).

56. M. A. Fullana, A. Albajes-Eizagirre, C. Soriano-Mas, B. Vervliet, N. Cardoner, O. Benet, J. Radua, B. J. Harrison, Fear extinction in the human brain: A meta-analysis of fMRI studies in healthy participants. *Neurosci. Biobehav. Rev.* **88**, 16–25 (2018).

57. M. A. McDannald, F. Lucantonio, K. A. Burke, Y. Niv, G. Schoenbaum, Ventral striatum and orbitofrontal cortex are both required for model-based, but not model-free, reinforcement learning. *J. Neurosci.* **31**, 2700–2705 (2011).

58. J. A. Gottfried, R. J. Dolan, Human orbitofrontal cortex mediates extinction learning while accessing conditioned representations of value. *Nat. Neurosci.* **7**, 1144–1152 (2004).

59. P. H. Rudebeck, R. C. Saunders, A. T. Prescott, L. S. Chau, E. A. Murray, Prefrontal mechanisms of behavioral flexibility, emotion regulation and value updating. *Nat. Neurosci.* **16**, 1140–1145 (2013).

60. B. B. Averbeck, V. D. Costa, Motivational neural circuits underlying reinforcement learning. *Nat. Neurosci.* **20**, 505–512 (2017).

61. V. D. Costa, O. Dal Monte, D. R. Lucas, E. A. Murray, B. B. Averbeck, Amygdala and ventral striatum make distinct contributions to reinforcement learning. *Neuron* **92**, 505–517 (2016).

62. J. Courtin, Y. Bitterman, S. Müller, J. Hinz, K. M. Hagihara, C. Müller, A. Lüthi, A neuronal mechanism for motivational control of behavior. *Science* **375**, eabg7277 (2022).

63. B. Hsueh, R. Chen, Y. Jo, D. Tang, M. Raffiee, Y. S. Kim, M. Inoue, S. Randles, C. Ramakrishnan, S. Patel, D. K. Kim, T. X. Liu, S. H. Kim, L. Tan, L. Mortazavi, A. Cordero, J. Shi, M. Zhao, T. T. Ho, A. Crow, A.-C. W. Yoo, C. Raja, K. Evans, D. Bernstein, M. Zeineh, M. Goubran, K. Deisseroth, Cardiogenic control of affective behavioural state. *Nature* **615**, 292–299 (2023).

64. I. Kauvar, E. B. Richman, T. X. Liu, C. Li, S. Vesuna, A. Chibukhchyan, L. Yamada, A. Fogarty, E. Solomon, E. Y. Choi, L. Mortazavi, G. C. L. Kung, P. Mukunda, C. Raja, D. Gil-Hernández, K. Patron, X. Zhang, J. Brawer, S. Wrobel, Z. Lusk, D. Lyu, A. Mitra, L. Hack, L. Luo, L. Grosenick, P. van Roessel, L. M. Williams, B. D. Heifets, J. M. Henderson, J. A. McNab, C. I. Rodríguez, V. Buch, P. Nuyujukian, K. Deisseroth, Conserved brain-wide emergence of emotional response from sensory experience in humans and mice. *Science* **388**, eadt3971 (2025).

65. J. D. Ramsey, S. J. Hanson, C. Hanson, Y. O. Halchenko, R. A. Poldrack, C. Glymour, Six problems for causal inference from fMRI. *Neuroimage* **49**, 1545–1558 (2010).

66. M. Koenigs, E. D. Huey, V. Raymont, B. Cheon, J. Solomon, E. M. Wassermann, J. Grafman, Focal brain damage protects against post-traumatic stress disorder in combat veterans. *Nat. Neurosci.* **11**, 232–237 (2008).

67. N. Koen, J. Fourie, D. Terburg, R. Stoop, B. Morgan, D. J. Stein, J. van Honk, Translational neuroscience of basolateral amygdala lesions: Studies of urbach-wiethe disease. *J. Neurosci. Res.* **94**, 504–512 (2016).

68. J. Li, D. Schiller, G. Schoenbaum, E. A. Phelps, N. D. Daw, Differential roles of human striatum and amygdala in associative learning. *Nat. Neurosci.* **14**, 1250–1252 (2011).

69. H. R. Siebner, K. Funke, A. S. Aberra, A. Antal, S. Bestmann, R. Chen, J. Classen, M. Davare, V. Di Lazzaro, P. T. Fox, M. Hallett, A. N. Karabanov, J. Kesselheim, M. Malling Beck, G. Koch, D. Liebetanz, S. Meunier, C. Miniussi, W. Paulus, A. V. Peterchev, T. Popa, M. C. Ridding, A. Thielscher, U. Ziemann, J. C. Rothwell, Y. Ugawa, Transcranial magnetic stimulation of the brain: What is stimulated?—A consensus and critical position paper. *Clin. Neurophysiol.* **140**, 59–97 (2022).

70. M. Wischnewski, I. Alekseichuk, A. Opitz, Neurocognitive, physiological, and biophysical effects of transcranial alternating current stimulation. *Trends Cogn. Sci.* **27**, 189–205 (2023).

71. T. Nandi, B. R. Kop, K. B. Pauly, C. J. Stagg, L. Verhagen, The relationship between parameters and effects in transcranial ultrasonic stimulation. *Brain Stimul.* **17**, 1216–1228 (2024).

72. K. Murphy, E. Fouragnan, The future of transcranial ultrasound as a precision brain interface. *PLOS Biol.* **22**, e3002884 (2024).

73. B. R. Kop, L. de Jong, B. P. Kim, H. E. M. den Ouden, L. Verhagen, Parameter optimisation for mitigating somatosensory confounds during transcranial ultrasonic stimulation. *Brain Stimul.* **18**, 1224–1236 (2025).

74. W. Legon, S. Adams, P. Bansal, P. D. Patel, L. Hobbs, L. Ai, J. K. Mueller, G. Meekins, B. T. Gillick, A retrospective qualitative report of symptoms and safety from transcranial focused ultrasound for neuromodulation in humans. *Sci. Rep.* **10**, 5573 (2020).

75. B. R. Kop, Y. S. Oghli, T. C. Grippe, T. Nandi, J. Lefkes, S. W. Meijer, S. Farboud, M. Engels, M. Hamani, M. Null, A. Radetz, U. Hassan, G. Darmani, A. Chetverikov, H. E. M. den Ouden, T. O. Bergmann, R. Chen, L. Verhagen, Auditory confounds can drive online effects of transcranial ultrasonic stimulation in humans. *eLife* **12**, RP88762 (2024).

76. K. R. Murphy, J. S. Farrell, J. Bendig, A. Mitra, C. Luff, I. A. Stelzer, H. Yamaguchi, C. C. Angelakos, M. Choi, W. Bian, T. DiIanni, E. M. Pujol, N. Matosevich, R. Airan, B. Gaudillière, E. E. Konofagou, K. Butts-Pauly, I. Soltesz, L. de Lecea, Optimized ultrasound neuromodulation for non-invasive control of behavior and physiology. *Neuron* **112**, 3252–3266.e5 (2024).

77. H.-J. Kim, T. T. Phan, K. Lee, J. S. Kim, S.-Y. Lee, J. M. Lee, J. Do, D. Lee, S.-P. Kim, K. P. Lee, J. Park, C. J. Lee, J. M. Park, Long-lasting forms of plasticity through patterned ultrasound-induced brainwave entrainment. *Sci. Adv.* **10**, eadk3198 (2024).

78. M. Mohammadjavadi, R. T. Ash, G. H. Glover, K. B. Pauly, Optimization of MR acoustic radiation force imaging (MR-ARFI) for human transcranial focused ultrasound. *Magn. Reson. Med.* **94**, 1060–1071 (2025).

79. J. E. Dunsmoor, Y. Niv, N. Daw, E. A. Phelps, Rethinking extinction. *Neuron* **88**, 47–63 (2015).

80. D. Schiller, M.-H. Monfils, C. M. Raio, D. C. Johnson, J. E. LeDoux, E. A. Phelps, Preventing the return of fear in humans using reconsolidation update mechanisms. *Nature* **463**, 49–53 (2010).

81. T. Agren, J. Engman, A. Frick, J. Björkstrand, E.-M. Larsson, T. Furmark, M. Fredrikson, Disruption of reconsolidation erases a fear memory trace in the human amygdala. *Science* **337**, 1550–1552 (2012).

82. M.-F. Marin, J. A. Camprodon, D. D. Dougherty, M. R. Milad, Device-based brain stimulation to augment fear extinction: Implications for PTSD treatment and beyond. *Depress. Anxiety* **31**, 269–278 (2014).

83. R. J. Koek, J. Avecillas-Chasin, S. E. Krahl, J. WY. Chen, D. L. Sultzer, A. D. Kulick, M. A. Mandelkern, M. Malpetti, H. L. Gordon, H. N. Landry, E. H. Einstein, J.-P. Langevin, Deep brain stimulation of the amygdala for treatment-resistant combat post-traumatic stress disorder: Long-term results. *J. Psychiatr. Res.* **175**, 131–139 (2024).

84. W. Legon, T. F. Sato, A. Opitz, J. Mueller, A. Barbour, A. Williams, W. J. Tyler, Transcranial focused ultrasound modulates the activity of primary somatosensory cortex in humans. *Nat. Neurosci.* **17**, 322–329 (2014).

85. A. Fomenko, K.-H. S. Chen, J.-F. Nankoo, J. Saravanamuttu, Y. Wang, M. El-Baba, X. Xia, S. S. Seerala, K. Hynynen, A. M. Lozano, R. Chen, Systematic examination of low-intensity ultrasound parameters on human motor cortex excitability and behaviour. *eLife* **9**, e54497 (2020).

86. C. Liu, K. Yu, X. Niu, B. He, Transcranial focused ultrasound enhances sensory discrimination capability through somatosensory cortical excitation. *Ultrasound Med. Biol.* **47**, 1356–1366 (2021).

87. L. D. de Voogd, J. W. Kanen, D. A. Neville, K. Roelofs, G. Fernández, E. J. Hermans, Eye-movement intervention enhances extinction via amygdala deactivation. *J. Neurosci.* **38**, 8694–8706 (2018).

88. L. D. de Voogd, E. A. Phelps, A cognitively demanding working-memory intervention enhances extinction. *Sci. Rep.* **10**, 7020 (2020).

89. F. Faul, E. Erdfelder, A.-G. Lang, A. Buchner, G*Power 3: A flexible statistical power analysis program for the social, behavioral, and biomedical sciences. *Behav. Res. Methods* **39**, 175–191 (2007).

90. A. N. Zsido, N. Arato, O. Inhof, J. Janszky, G. Darnai, Short versions of two specific phobia measures: The snake and the spider questionnaires. *J. Anxiety Disord.* **54**, 11–16 (2018).

91. E. S. Dan-Glauser, K. R. Scherer, The Geneva affective picture database (GAPED): A new 730-picture database focusing on valence and normative significance. *Behav. Res. Methods* **43**, 468 (2011), 477.

92. J. Peirce, J. R. Gray, S. Simpson, M. MacAskill, R. Höchenberger, H. Sogo, E. Kastman, J. K. Lindeløv, PsychoPy2: Experiments in behavior made easy. *Behav. Res. Methods* **51**, 195–203 (2019).

93. S. Yoo, D. R. Mittelstein, R. C. Hurt, J. Lacroix, M. G. Shapiro, Focused ultrasound excites cortical neurons via mechanosensitive calcium accumulation and ion channel amplification. *Nat. Commun.* **13**, 493 (2022).

94. G. Darmani, H. Ramezanpour, C. Sarica, R. Annirood, T. Grippe, J.-F. Nankoo, A. Fomenko, B. Santyr, K. Zeng, A. Vetkas, N. Samuel, B. Davidson, A. Fasano, M. Lankarany, S. K. Kalia, S. Pichardo, A. M. Lozano, R. Chen, Individualized non-invasive deep brain stimulation of the basal ganglia using transcranial ultrasound stimulation. *Nat. Commun.* **16**, 2693 (2025).

95. S. Bao, H. Kim, N. B. Shettigar, Y. Li, Y. Lei, Personalized depth-specific neuromodulation of the human primary motor cortex via ultrasound. *J. Physiol.* **602**, 933–948 (2024).

96. J.-F. Aubry, D. Attali, M. E. Schafer, E. Fouragnan, C. F. Caskey, R. Chen, G. Darmani, E. J. Bubrick, J. Sallet, C. R. Butler, C. J. Stagg, M. C. Klein-Flügge, S.-S. Yoo, C. K. Holland, B. Treeby, E. Martin, L. Verhagen, K. Butts Pauly, ITRUSST consensus on biophysical safety for transcranial ultrasound stimulation. *Brain Stimul.* **18**, 1896–1905 (2025).

97. E. A. Murray, A. Izquierdo, Orbitofrontal cortex and amygdala contributions to affect and action in primates. *Ann. N. Y. Acad. Sci.* **1121**, 273–296 (2007).

98. Z. M. Saygin, D. Kliemann, J. E. Iglesias, A. J. W. van der Kouwe, E. Boyd, M. Reuter, A. Stevens, K. Van Leemput, A. McKee, M. P. Frosch, B. Fischl, J. C. Augustinack, High-resolution magnetic resonance imaging reveals nuclei of the human amygdala: Manual segmentation to automatic atlas. *Neuroimage* **155**, 370–382 (2017).

99. J. E. Iglesias, J. C. Augustinack, K. Nguyen, C. M. Player, A. Player, M. Wright, N. Roy, M. P. Frosch, A. C. McKee, L. L. Wald, B. Fischl, K. Van Leemput, A computational atlas of the hippocampal formation using ex vivo, ultra-high resolution MRI: Application to adaptive segmentation of in vivo MRI. *Neuroimage* **115**, 117–137 (2015).

100. B. Fischl, FreeSurfer. *NeuroImage* **62**, 774–781 (2012).

101. B. E. Treeby, B. T. Cox, Modeling power law absorption and dispersion for acoustic propagation using the fractional Laplacian. *J. Acoust. Soc. Am.* **127**, 2741–2748 (2010).

102. K. R. Murphy, T. Nandi, B. Kop, T. Osada, M. Lueckel, W. A. N'Djin, K. A. Caulfield, A. Fomenko, H. R. Siebner, Y. Ugawa, L. Verhagen, S. Bestmann, E. Martin, K. Butts Pauly, E. Fouragnan, T. O. Bergmann, A practical guide to transcranial ultrasonic stimulation from the IFCN-endorsed ITRUSST consortium. *Clin. Neurophysiol.* **171**, 192–226 (2025).

103. D. C. Knight, H. T. Nguyen, P. A. Bandettini, The role of the human amygdala in the production of conditioned fear responses. *Neuroimage* **26**, 1193–1200 (2005).

104. C. S. Inman, K. R. Bijanki, D. I. Bass, R. E. Gross, S. Hamann, J. T. Willie, Human amygdala stimulation effects on emotion physiology and emotional experience. *Neuropsychologia* **145**, 106722 (2020).

105. S. R. Green, P. A. Kragel, M. E. Fecteau, K. S. LaBar, Development and validation of an unsupervised scoring system (Autonomate) for skin conductance response analysis. *Int. J. Psychophysiol.* **91**, 186–193 (2014).

106. W. Boucsein, D. C. Fowles, S. Grimnes, G. Ben-Shakhar, W. T. Roth, M. E. Dawson, D. L. Filion, Society for Psychophysiological Research Ad Hoc Committee on Electrodermal Measures, Publication recommendations for electrodermal measurements. *Psychophysiology* **49**, 1017–1034 (2012).

107. R. Sjouwerman, T. B. Lonsdorf, Latency of skin conductance responses across stimulus modalities. *Psychophysiology* **56**, e13307 (2019).

108. D. Bates, M. Mächler, B. Bolker, S. Walker, Fitting linear mixed-effects models using lme4. *J. Stat. Softw.* **67**, 1–48 (2015).

109. J. Fox, S. Weisberg, B. Price, car: Companion to Applied Regression (2001); https://doi.org/10.32614/CRAN.package.car.

110. J. Fox, S. Weisberg, B. Price, D. Adler, D. Bates, G. Baud-Bovy, B. Bolker, S. Ellison, D. Firth, M. Friendly, G. Gorjanc, S. Graves, R. Heiberger, P. Krivitsky, R. Laboissiere, M. Maechler, G. Monette, D. Murdoch, H. Nilsson, D. Ogle, B. Ripley, T. Short, W. Venables, S.

Walker, D. Winsemius, A. Zeileis, R-Core, car: Companion to Applied Regression, version 3.1-3 (2024); https://cran.r-project.org/web/packages/car/index.html.

111. R. V. Lenth, B. Banfai, B. Bolker, P. Buerkner, I. Giné-Vázquez, M. Herve, M. Jung, J. Love, F. Miguez, J. Piaskowski, H. Riebl, H. Singmann, emmeans: Estimated Marginal Means, aka Least-Squares Means, version 1.11.1 (2025); https://cran.r-project.org/web/packages/emmeans/index.html.

112. A. M. Winkler, G. R. Ridgway, M. A. Webster, S. M. Smith, T. E. Nichols, Permutation inference for the general linear model. *Neuroimage* **92**, 381–397 (2014).

113. A. Gelman, D. B. Rubin, Inference from iterative simulation using multiple sequences. *Statist. Sci.* **7**, 457–472 (1992).

114. C. R. Quirk, I. Zutshi, S. Srikanth, M. L. Fu, N. Devico Marciano, M. K. Wright, D. F. Parsey, S. Liu, R. E. Siretskiy, T. L. Huynh, J. K. Leutgeb, S. Leutgeb, Precisely timed theta oscillations are selectively required during the encoding phase of memory. *Nat. Neurosci.* **24**, 1614–1627 (2021).

115. M. E. Bouton, R. F. Westbrook, K. A. Corcoran, S. Maren, Contextual and temporal modulation of extinction: Behavioral and biological mechanisms. *Biol. Psychiatry* **60**, 352–360 (2006).

116. D. Attali, T. Tiennot, M. Schafer, E. Fouragnan, J. Sallet, C. F. Caskey, R. Chen, G. Darmani, E. J. Bubrick, C. Butler, C. J. Stagg, M. Klein-Flügge, L. Verhagen, S.-S. Yoo, K. B. Pauly, J.-F. Aubry, Three-layer model with absorption for conservative estimation of the maximum acoustic transmission coefficient through the human skull for transcranial ultrasound stimulation. *Brain Stimul.* **16**, 48–55 (2023).
